# Comparative genomics reveals diversity and taxonomic relationships among *Clostridioides difficile* phages

**DOI:** 10.1128/spectrum.01431-25

**Published:** 2025-11-12

**Authors:** Mohimenul Haque Rolin, Shakhinur Islam Mondal, Daniyal Karim, Nurnabi Azad Jewel, Tahsin Khan, Mustafizur Rahman, Arzuba Akter

**Affiliations:** 1 Department of Genetic Engineering and Biotechnology, Shahjalal University of Science and Technology 113074, Sylhet, Bangladesh; 2 Parasites and Microbes Programme, Wellcome Sanger Institute, Hinxton, United Kingdom; 3 Genome Centre, Infectious Diseases Division, icddr,b, Dhaka, Bangladesh; 4 Virology Laboratory, Infectious Diseases Division, icddr,b 56291 , Dhaka, Bangladesh; 5 Department of Biochemistry and Molecular Biology, Shahjalal University of Science and Technology 113074, Sylhet, Bangladesh; Emory University School of Medicine, Atlanta, Georgia, USA

**Keywords:** *Clostridioides difficile*, bacteriophage, endolysins, comparative genomics, taxonomic classification

## Abstract

*Clostridioides difficile* is associated with life-threatening antibiotic-associated diarrhea, colitis, and toxin-mediated infections. While antibiotics are the primary treatment against *C. difficile* infections, increasing resistance necessitates alternatives. Bacteriophages and bacteriophage-derived proteins, such as endolysins, hold promise as potential solutions. Understanding phage biology at the genomic level is crucial for their therapeutic use. We conducted a comparative genomic analysis of 44 *C*. *difficile* phage genomes from public databases, examining both whole-genome and proteome levels and grouping them by shared protein content. Relationships within each group were observed, and core and highly conserved genes were identified. Using genome and proteome phylogeny, average nucleotide identity, and core gene identification, we proposed an updated taxonomic classification. Nine distinct clusters were identified, without any singleton. Cluster members exhibited similar genome architecture, genome sizes, GC content, number of coding sequences, presence of core genes, and high nucleotide identity. Additionally, we propose 23 new genera, three families, and the elevation of currently assigned genera to subfamilies. The lytic module proteins, endolysins, and holins were also characterized, revealing four distinct endolysin organizations with diverse domain architectures. Notably, the amidase_3 and LysM domains were highly conserved and subjected to purifying selection within the *C. difficile* phage genomes. Our extensive comparative analyses of *C. difficile* phage genomes provide valuable genomic insights into the current understanding of the phages. The taxonomic analysis could refine the classification scheme for these phages and facilitate the future categorization of newly isolated *C. difficile* phage genomes.

**IMPORTANCE:**

*Clostridioides difficile* is a significant healthcare concern due to its role in causing severe infections and its increasing resistance to antibiotics. Phages present a promising alternative to antibiotics for combating bacterial pathogens, including *C. difficile*. Our study is the first comprehensive comparative genomic analysis of *C. difficile* phage genomes. We analyzed all available *C. difficile* phage genomes and clustered them into genetically distinct groups based on protein sharing. We also propose major taxonomic revisions to facilitate the accurate classification of *C. difficile* phages. Additionally, we investigated the lytic module of these phages, revealing diverse endolysin organizations and strong purifying selection acting on the catalytic and cell wall binding domains. Our findings contribute valuable insights into the biology of *C. difficile* phages and provide a framework that can aid in the classification and genomic analysis of newly isolated *C. difficile* phages in the future.

## INTRODUCTION

*Clostridioides difficile* is a gram-positive, spore-forming anaerobic bacterium (1). It is a significant causative agent of nosocomial antibiotic-associated diarrhea, particularly in developed countries. Prevalent in the mammalian gastrointestinal tract, *C. difficile* can lead to toxin-mediated *C. difficile* infections (CDIs), which manifest as a spectrum of symptoms from mild diarrhea to severe conditions such as pseudomembranous colitis, with potentially fatal outcomes. In the United States alone, *C. difficile* is responsible for over 500,000 infections annually, resulting in approximately 29,000 deaths and imposing a substantial financial burden estimated at $1–3 billion (2). CDI is linked to three well-characterized toxins—toxin A, toxin B, and binary toxin. While the former two toxins are considered primary virulence factors for CDI, *C. difficile* binary toxin has been associated with increased severity of *C. difficile* infection (3, 4). *C. difficile* is commonly found in the gut microbiota of healthy individuals without causing any symptoms of the disease (5, 6). However, disruptions in the gut microbiota, such as those caused by antibiotic administration or malnutrition, can predispose individuals to CDI. This imbalance in microbial composition, known as dysbiosis, can elevate concentrations of *C. difficile* toxins, facilitating colonization and the formation of biofilms in the gut. These processes contribute to the progression of severe disease states associated with CDI (6, 7). Furthermore, spores of *C. difficile* are resistant to antibiotics. Following cessation of antibiotic therapy, residual spores germinate in the altered gut, leading to recurrent CDI (8, 9). Fecal microbiota transplant (FMT) has been promising to prevent recurrent infections. However, several risks are involved, including the potential transfer of undesired microbes and uncertainties regarding its long-term health impact (9, 10). Consequently, there is an urgent need for alternative therapeutic strategies to address recurrent *C. difficile* infections beyond FMT.

Bacteriophages or phages are viruses that infect and ultimately kill bacterial hosts (11). Phages are adapted to two lifestyles—lytic and lysogenic. Strictly lytic phages are known as virulent. In contrast, temperate phages in normal conditions are lysogenic. However, they can switch to the lytic cycle in times of stress. At the end of the lytic cycle, phages lyse bacterial cell walls, leading to cell death (12). Phage therapy utilizes this property of phages, which involves the administration of phages to kill target bacteria. The major advantage of phages is their high specificity toward target bacteria while sparing beneficial bacteria (11). This intriguing feature makes phages an attractive alternative to treat CDI or recurrent CDI. Different *C. difficile* phages have been assessed for their lytic potential in multiple *in vitro* assays (13–15). Phage cocktails with three or four different phages showed more lytic efficacy compared to single phage treatments. However, rebound to lysogens was a common occurrence observed in *C. difficile* phages due to their temperate nature (9). Creation of strictly lytic phages using a genetic engineering approach has been attempted, but the result was similar to wild type with recombinant lytic phages reverting to the lysogenic state (16). Phage-derived proteins such as endolysins offer a promising alternative to using whole phages (17, 18). Phages use endolysin to hydrolyze bacterial cell walls from within, at the end of their lytic cycle (19). Several *C. difficile* phage endolysins have been cloned, characterized, and assessed for their lytic efficacy *in vitro* (9, 20).

With the ever-increasing availability of fully sequenced phage genomes, *in silico* studies can elucidate phage biology, evolution, and host-phage relationships, thus aiding in identifying therapeutically important phages and phage-derived proteins. Whole-genome comparative analysis has been performed on phages infecting *Mycobacterium*, *Acinetobacter*, *Staphylococcus*, *Pseudomonas*, *Salmonella*, *Vibrio cholerae*, and Alphaproteobacteria (21–28). These studies revealed vast diversity among the phage population. Phage genomes were grouped into multiple clusters based on their relatedness, with those displaying no similarity to other phage genomes designated as “singletons.” Moreover, proteins were clustered into similar groups, called “phams,” while unique proteins lacking any significant relationship with other proteins were characterized as “orphams.” Using various computational approaches such as nucleotide identity, gene sharing, single gene, and network-based phylogeny, taxonomic proposals were suggested in studies involving *Acinetobacter* phages (23). The comparative genomics study on *Vibrio cholerae* phages explored the therapeutic potential of the *V. cholerae* phages, showcasing a systematic approach for screening therapeutic phages in cholera and other bacterial diseases (27). Comprehensive reviews of *C. difficile* phages have focused on genomic characteristics, utilizing phylogenetic analyses, genome alignments, and modular organization to detail functional regions, including DNA packaging, structural components, lysis, and lysogeny control modules (29). Recent advancements, however, emphasize the importance of whole-genome-based methods for defining taxonomic relationships, especially as more *C. difficile* phage genomes are sequenced, highlighting the need for more comprehensive genomic analyses (30). Such expanded studies could elucidate the relationship among the *C. difficile* phages, their genomic diversity, and classification, and identify potential therapeutic options.

In this study, we conducted a comprehensive analysis of all available sequenced *C. difficile* phage genomes. Genomic comparisons were carried out utilizing different computational approaches. The genomes were grouped into multiple clusters and subclusters, and each cluster was thoroughly investigated. Furthermore, we established genus (subcluster) and family-level relationships among the phages. Finally, we examined the lysis module of CD phages, elucidating the diversity of endolysin and holin proteins.

## MATERIALS AND METHODS

### Genome acquisition annotation

Complete genomes of *C. difficile* (CD) phages were obtained from the INPHARED database (last accessed 01 March 2024) (https://github.com/RyanCook94/inphared) (31). CD-HIT was used to check for any duplication of phage genomes with 1.0 identity coverage (-c) and a word size of 10 (*n* = 10) (32). The genomes were then re-annotated using Pharokka (version 1.7.5), with Prodigal being used for gene calling in meta mode and PHROGs database for functional annotations (33). The “terminase large subunit” gene was used as the reference starting point, and genome reorientation was performed using the Dnaapler program within the Pharokka pipeline to ensure consistent gene start positions across genomes (34). Phage lifestyle prediction was carried out using BacPhlip and PhaTyp (35, 36).

### Genome clustering

Phammseqs was used to assign proteins of all genomes to phage protein families or phams with pangenome option (-p) enabled. For clustering the proteins into similar groups of proteins or phams, 35% amino acid identity and 80% coverage were used (37). Based on shared phams among the genomes, CD phages were clustered using PhamClust using a clustering threshold of 0.3 (38). A tab-separated (TSV) file, mapping genomes to phams, and translations obtained from Phammseqs’ pangenome result were used as the input. VIRIDIC was used to calculate the intergenomic similarity of the phage genomes in default parameters (39). A 70% average nucleotide identity (ANI) cutoff value was used to assign cluster members into subclusters. Comparative genome maps were generated using gggenomes (version 1.0.0) (40). All vs all protein blasts were conducted to generate links among the genomes at an e-value of 1e−05.

### Pangenome analysis

Initially, pangenome analysis was conducted using Pirate at 30% identity (41). For identifying core genes within cluster members, 35% identity and 50% coverage were used. However, in the case of inter-cluster core gene identification, a less stringent identity threshold of 30% was used. Additionally, CoreGenes 5.0 was utilized to identify the core genes among the phage genomes using the following options: bidirectional best hit, paralogs enabled, and an e-value of 1e−05 (42).

### Phylogeny and taxonomy

The VICTOR web service (https://ggdc.dsmz.de/victor.php) was used to perform whole-genome-based phylogenetic analysis (43). This method classifies and creates a phylogeny of prokaryotic viruses based on their genome. All the nucleotide sequences were compared pairwise using the Genome-BLAST Distance Phylogeny (GBDP) method, which is recommended for prokaryotic viruses. The resulting intergenomic distances were used to create a balanced minimum evolution tree with branch support via FASTME. The tree was created for each of the formulas D0, D4, and D6, respectively. Branch support was inferred from 100 pseudo-bootstrap replicates each. The trees were rooted at the midpoint and visualized with ggtree. To estimate taxon boundaries at the species, genus, and family levels, the OPTSIL program was used with the recommended clustering thresholds and an *F* value of 0.5 (fraction of links required for cluster fusion). ViPTree server was used to generate proteomic trees of viral sequences based on genome-wide sequence similarities (44). The analysis was performed in both query mode and reference mode. dsDNA viruses with prokaryotes as hosts were selected as references. The resulting trees were visualized and annotated in iTOL (45). Metadata for manual annotation was retrieved from Virus-Host DB (46). Additionally, single-gene phylogenetic trees were constructed utilizing the ETE3 pipeline in the genome.jp server, using amino acid sequences of the corresponding genes. Alignment and phylogenetic reconstructions were performed using the function “build” of ETE3 3.1.2 as implemented on the GenomeNet (https://www.genome.jp/tools/ete/) (47). Alignment was performed with MAFFT version 6.861b with the default options (48). The resulting alignment was cleaned using the gappyout algorithm of trimAl version 1.4.rev6 (49). A Maximum likelihood (ML) tree was inferred using IQ-TREE 1.5.5, run with ModelFinder and tree reconstruction (50). Tree branches were tested by Shimodaira–Hasegawa-like approximate likelihood ratio test (SH-like aLRT) with 1,000 replicates. The resulting trees were rooted at the midpoint, annotated, and visualized in iTOL.

### Lysis module

For extracting the endolysins encoded by the CD phages, curated sequences of “bona fide” endolysins were used as the reference database for Diamond (51). A BLASTp identity of 30% was used for extracting endolysin sequences using Diamond (52). However, this method failed to extract all endolysins encoded by the CD phages. Thus, Phammseqs assigned phams that contained endolysins were also identified using “endolysin” as the keyword. A single representative sequence was included in the subsequent analyses for genomes that encode multiple endolysins of the same domain. Endolysin domains were identified using phmmer (53). Phylogenetic analysis was carried out by methods discussed in the previous section. Similarly, phams consisting of holin sequences were also identified, using “holin” as the keyword. Domain identification and phylogenetic analysis were carried out in the same way as for endolysins. To examine if endolysin and holin encoding genes went through selection pressure, analyses were carried out on selected phams of endolysins and holins. In the case of both genes, stop codons were removed and aligned with the codon-aware sequence alignment program, MACSE (54). The alignment was cleaned using trimAl with the gappyout algorithm. Datamonkey web server was utilized for selection pressure analysis using the following programs: GARD was used to take recombination event into account prior to selection pressure analysis using FEL, FUBAR, MEME, and SLAC with a *P* value threshold of 0.1 (55–59). Sites under positive and negative selection, detected by each model, were compared and grouped in Venn diagrams using the Venn diagram tool in the Bioinformatics and Evolutionary Genomics platform (http://bioinformatics.psb.ugent.be/webtools/Venn/).

### Comparison with the uncultivated virus data set

The uncultivated viral genome data set, IMG/VR v4, containing high-confidence genomes (*n* = 5,576,197), was downloaded (60). To search for similar sequences, representative genomes from each cluster, selected based on the highest average proteomic equivalence quotient (PEQ) score, were used. BLASTn searches were performed with each representative genome as the query against the IMG/VR databases, using the following parameters: “-task megablast -evalue 1e-5 -outfmt 6” (61). The BLAST results were filtered for ≥50% identity and ≥50% query coverage, and metadata for the matched genomes were extracted. Additionally, all available metadata for *C. difficile* phage genomes were collected. Only high-quality genome sequences, excluding RefSeq genomes, were annotated using Pharokka. Continuing our previous proteome-based approach, PhamClust was employed to identify similar genomes with a clustering threshold of 0.3 (38). vContact2 was executed with a combined data set containing viruses from *Caudoviricetes* excluding *Crassvirales* (*n* = 4,532) for reference and the high-quality CD genomes from IMG/VR (62). Finally, BacPhlip and PhaTyp were used to predict the lifestyles of the phages (35, 36).

## RESULTS

### Diversity of *C. difficile* phage genomes

**Fig 1:**
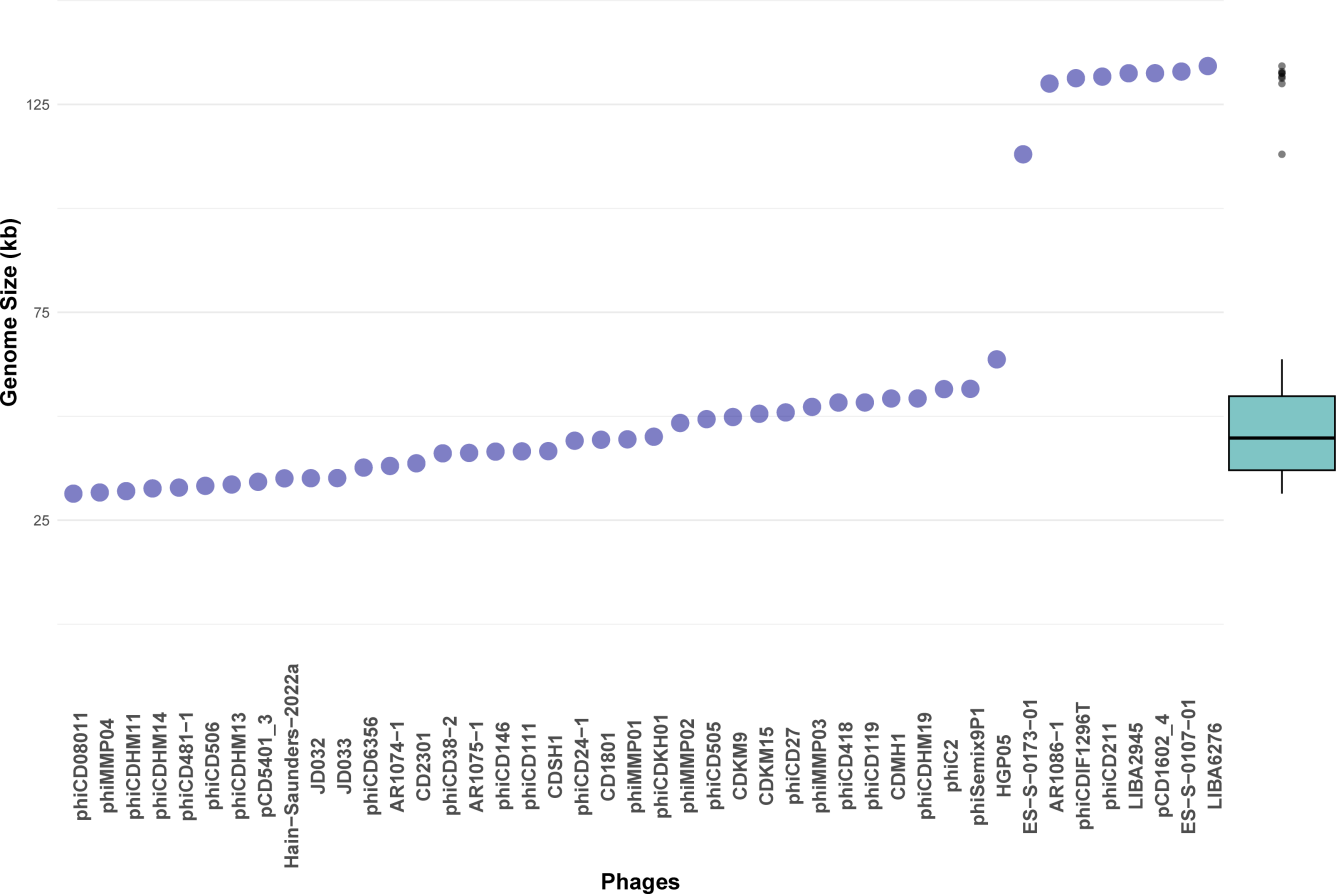
Scatterplot of genome length of the CD phages arranged in ascending order. Phages are ordered on the *X*-axis based on the genome length on the *Y*-axis. The box plot shows that most of the phage genomes are around 44 kbp, while eight genomes with >100 kbp genome length are clear outliers.

**Fig 2:**
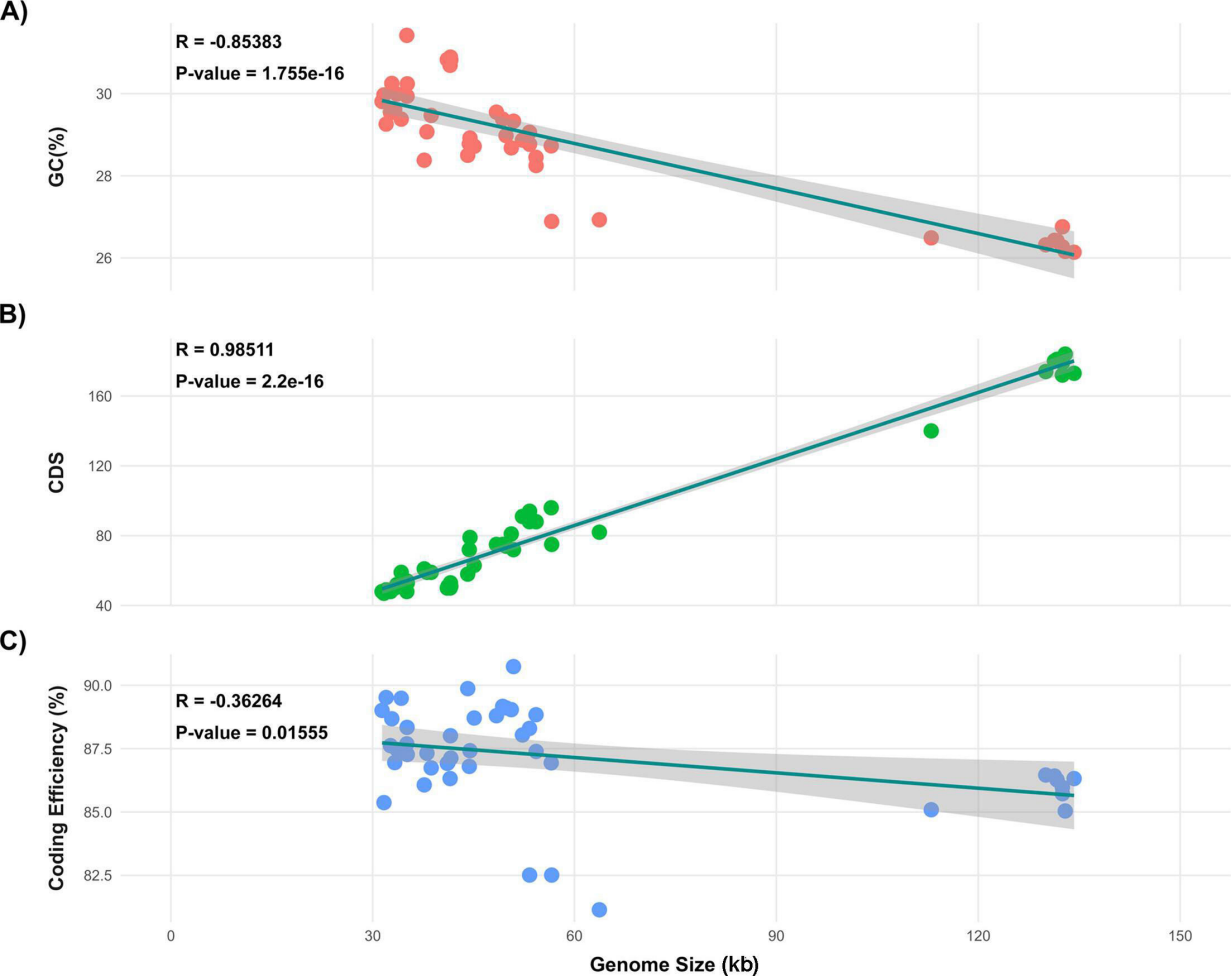
Correlation between genome size of the phages (*X*-axis) and three properties: GC content (%), number of coding sequences (CDS), and coding efficiency (%), each represented on the *Y*-axis. (**A**) Correlation between genome size and GC % was highly negative (*R* = −0.85383), while (**B**) genome size vs CDS was highly positive (*R* = 0.98511). (**C**) In contrast, the correlation between genome size and coding efficiency was slightly negative (*R* = −0.36264).

As of 1 March 2024, 44 sequenced genomes of CD phages were available in databases. These phages were found in various sources such as water, sewage slurry, hospitals, human fecal samples, and soil. All CD phages belong to the class *Caudoviricetes*. While their family is still unassigned, they are currently grouped into five distinct genera: *Sherbrookevirus* (*n* = 11), *Leicestervirus* (*n* = 6), *Colneyvirus* (*n* = 5), *Yongloolinvirus* (*n* = 4), and *Lubbockvirus* (*n* = 2). The remaining 16 phages are yet to be classified. The phage genomes exhibit significant diversity in length, ranging from 31 kb to 135 kb, with eight genomes exceeding 100 kb, while the rest measure around 44 kb. The scatterplot in Fig. 1 illustrates the length distribution of the phage genomes. The GC percentage ranges from 26% to 31%, and a highly negative correlation (Pearson’s *R* = −0.85383, *P* = 1.755e−13) exists between the GC percentage and the genome length. Conversely, there is a highly positive correlation (Pearson’s *R* = 0.98511, *P* = 2.2e−16) between the genome length and the number of coding sequences (CDS). In contrast, a slightly negative correlation (Pearson’s *R* = −0.36264, *P* = 0.01555) exists between the coding density and the genome length (Fig. 2). Searches with tRNAscan-se and ARAGORN revealed seven tRNAs in five of eight large phage genomes, except for LIBA6276, ES-S-0173-01, and pCD1602_4. Searches against the CARD and VFDB databases were conducted to identify resistance genes in phage genomes. Only one hit was found against the VFDB database. A hypothetical protein encoded by phage phiSemix9P1 had almost ~80% sequence identity with a CDT type toxin (cdtB), an ADP-ribosyltransferase binding component of *C. difficile CD196*. Searches against the CARD databases yielded only a few weak hits. In addition, Minced was used to identify CRISPR spacer sequences within phage genomes. Spacer sequences were identified in the following phage genomes: CDKM15, phiCD24-1, phiCDKH01, and phiSemix9P1. Table 1 summarizes the general characteristics and features of the phages.

**TABLE 1:** General information of *C. difficile* phages

Accession	Phages	Length	GC	CDS	Coding_density	Genus	tRNA
MF547662.1	LIBA6276	134,243	26.14	173	86.32	Unclassified	0
CP067347.1	ES-S-0107-01	132,924	26.16	184	85.04	Unclassified	2
CP069348.1	pCD1602_4	132,519	26.76	179	85.72	Unclassified	0
MF547663.1	LIBA2945	132,508	26.27	172	85.97	Unclassified	2
NC_029048.2	phiCD211	131,704	26.42	181	86.27	Unclassified	1
CP011970.1	phiCDIF1296T	131,326	26.43	180	86.42	Unclassified	1
OR397124.1	AR1086-1	130,030	26.32	174	86.46	Unclassified	1
CP067352.1	ES-S-0173-01	113,019	26.49	140	85.09	Unclassified	0
CP103976.1	HGP05	63,696	26.93	82	81.14	Unclassified	0
KX905163.1	phiSemix9P1	56,606	26.89	75	82.51	Unclassified	0
NC_009231.1	phiC2	56,538	28.73	96	86.94	*Yongloolinvirus*	0
NC_028996.1	phiCDHM19	54,295	28.25	88	87.39	*Lubbockvirus*	0
NC_024144.1	CDMH1	54,279	28.45	88	88.84	*Yongloolinvirus*	0
NC_007917.1	phiCD119	53,325	28.77	94	82.51	*Lubbockvirus*	0
MW512573.1	phiCD418	53,311	29.06	88	88.3	Unclassified	0
NC_028959.1	phiMMP03	52,261	28.87	91	88.04	*Yongloolinvirus*	0
NC_011398.1	phiCD27	50,930	29.33	72	90.74	*Colneyvirus*	0
NC_048643.1	CDKM15	50,605	28.68	81	89.04	*Colneyvirus*	0
NC_048642.1	CDKM9	49,822	28.98	74	89.11	*Colneyvirus*	0
NC_028764.1	phiCD505	49,316	29.38	75	89.17	*Colneyvirus*	0
NC_019421.1	phiMMP02	48,396	29.55	75	88.8	*Colneyvirus*	0
MN718463.1	phiCDKH01	45,089	28.72	63	88.71	Unclassified	0
NC_028883.1	phiMMP01	44,461	28.92	79	87.42	*Yongloolinvirus*	0
MW512570.1	CD1801	44,363	28.78	72	86.8	Unclassified	0
LN681534.1	phiCD24-1	44,129	28.5	58	89.87	Unclassified	0
KU057941.1	CDSH1	41,619	30.82	51	87.13	*Leicestervirus*	0
NC_028905.1	phiCD111	41,560	30.89	53	88.01	*Leicestervirus*	0
NC_028958.1	phiCD146	41,507	30.69	50	86.32	*Leicestervirus*	0
OQ703261.1	AR1075-1	41,195	30.84	51	86.95	*Leicestervirus*	0
NC_015568.1	phiCD38-2	41,090	30.83	50	86.92	*Leicestervirus*	0
MW512571.1	CD2301	38,695	29.47	59	86.74	*Sherbrookevirus*	0
OR397123.1	AR1074-1	38,059	29.07	59	87.32	Unclassified	0
NC_015262.1	phiCD6356	37,664	28.38	61	86.07	Unclassified	0
MT193276.1	JD033	35,144	30.24	53	87.27	*Sherbrookevirus*	0
MK473382.1	JD032	35,109	29.94	54	88.34	*Sherbrookevirus*	0
CP103806.1	Hain-Saunders-2022a	35,072	31.42	48	87.69	*Leicestervirus*	0
CP069347.1	pCD5401_3	34,243	29.38	59	89.49	*Sherbrookevirus*	0
NC_029116.1	phiCDHM13	33,596	29.99	52	87.47	*Sherbrookevirus*	0
NC_028838.1	phiCD506	33,274	29.61	50	86.95	*Sherbrookevirus*	0
NC_028951.1	phiCD481-1	32,846	30.25	49	88.68	*Sherbrookevirus*	0
NC_048665.1	phiCDHM14	32,651	29.54	48	87.62	*Sherbrookevirus*	0
NC_029001.1	phiCDHM11	32,000	29.26	49	89.52	*Sherbrookevirus*	0
NC_019422.1	phiMMP04	31,674	29.97	47	85.37	*Sherbrookevirus*	0
MW512572.1	phiCD08011	31,394	29.81	48	89.01	*Sherbrookevirus*	0

### Genomic comparison reveals nine clusters without any singleton

**Fig 3:**
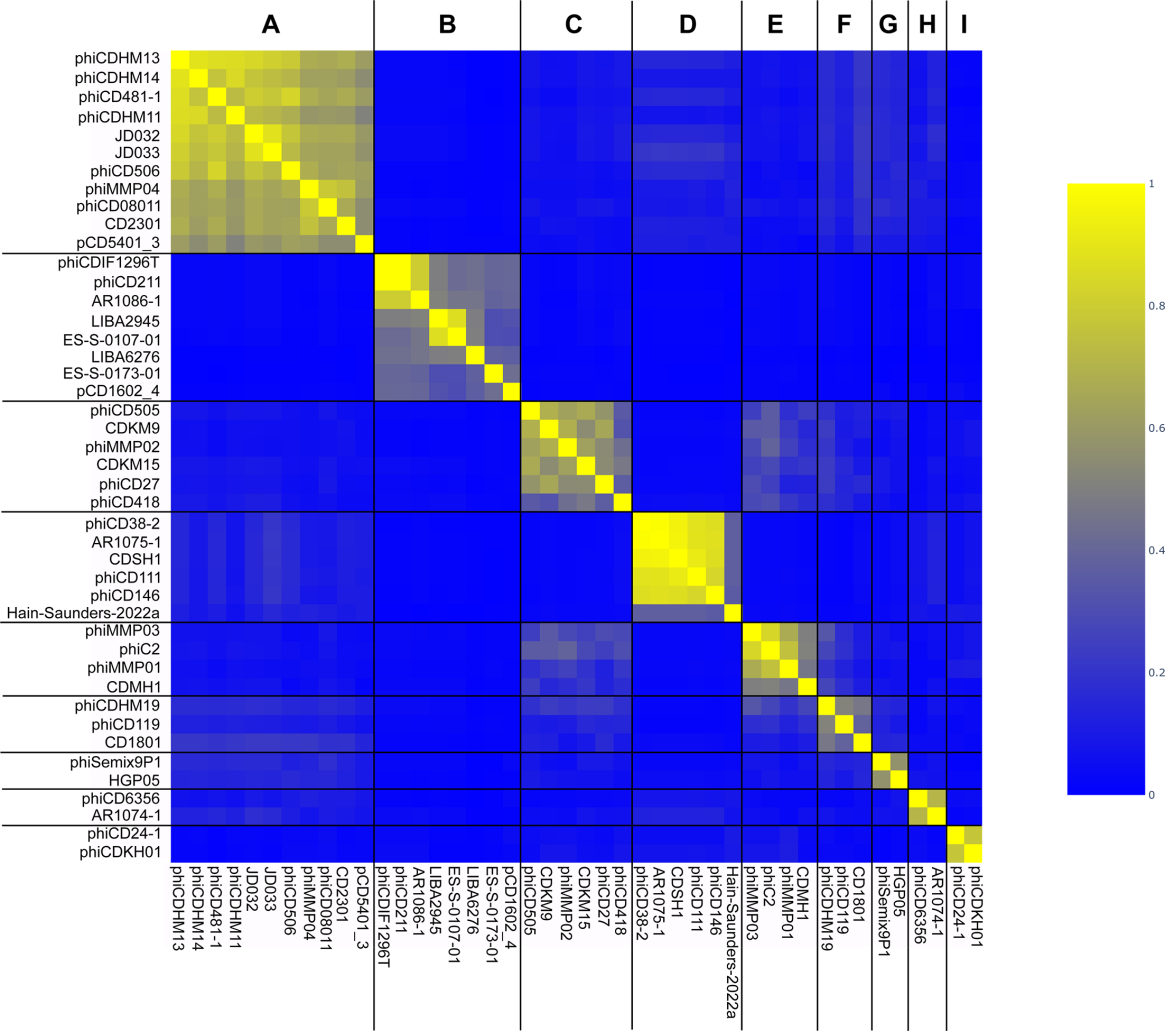
Heatmap of PEQ values among the CD phage genomes. Based on the PEQ values, 44 phage genomes were assigned to nine clusters. Assigned clusters (A to I) are shown on the top horizontal axis.

For clustering phage genomes, we used protein sharing as the main criterion. PhamClust computes a PEQ for each phage pair by assessing the amino acid sequence identity of genes shared between them. PEQ values indicate the percentage of gene sharing—0% value indicates no shared genes, while 100% indicates all genes shared at 100% identity. The 44 phage genomes formed nine distinct clusters (A to I) without any singleton (Fig. 3). Cluster-wise gene sharing percentages are included in Table S1. The same clustering organization was also observed based on whole genome vs whole-genome nucleotide identity visualized in the ANI heatmap (Fig. S1). Additionally, using a 70% ANI cutoff value, 21 subclusters (denoted as A1, A2, B1, etc.) were identified (Fig. S2 through S8). The 44 CD phages encode a total of 3,725 proteins. Proteins were assigned to 935 phams using Phammseqs at 35% mmseqs2 identity and 80% coverage. Among them, 312 (33.37%) were orphams, containing only one protein with no sequence similarity with other proteins. Phages phiCDIF1296T, phiCD38-2, phiMMP02, and phiMMP03 contain no orphams, while phiCD119, ES-S-0173-01, pCD1602_4, and ES-S-0107-01 contain a high number of orphams (>20) (Fig. S9).

Pangenome analysis of 44 genomes using Pirate with a 30% identity threshold identified a single core gene encoding an endolysin protein. Pirate defines a core gene as one that is present in at least 95% of the genomes in a population. However, given that our analysis involves only 44 genomes, we would consider a gene to be a core gene only if it is present in 100% of the genomes. Thus, manual curation revealed this gene’s absence in all analyzed genomes. Further investigation identified two endolysins of the same domains present within the CD2301 phage, potentially inflating the core gene designation by Pirate. Another analysis with Phammseqs identified a soft-core endolysin gene (defined by its presence in 95%–99% of genomes), further suggesting the absence of a consistent core gene. Finally, Coregenes 5.0 analysis corroborated these findings, identifying no core genes.

#### Cluster A

**Fig 4:**
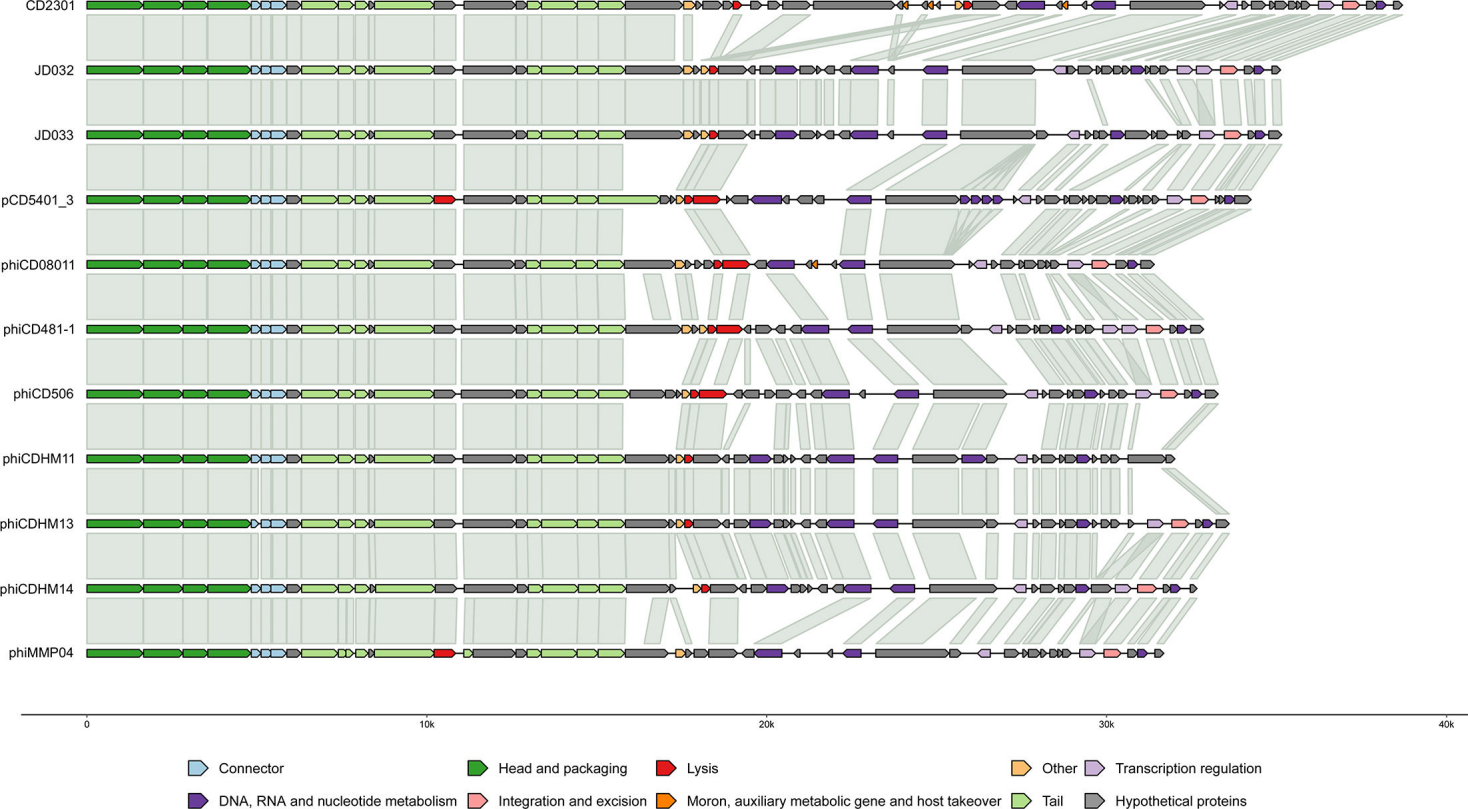
Genome comparison among cluster A phage genomes. The genomes were observed to be highly conserved. Specific modular organization is observed with high conservation visible across all modules. The genes are colored according to their functional categories as indicated in the legend.

Cluster A, the largest cluster with 11 genomes, consists of phages classified as *Sherbrookevirus* at the genus level. These phage genomes have a genome length ranging from 31 kbp to 38 kbp with a 29%–30% GC percentage. Within Cluster A, the phages encode 48–59 genes with an average coding density of 87%. Pangenome analysis using PIRATE identified a total of 85 gene families, with 25 core genes shared among the genomes of Cluster A phages (29%). These core genes are categorized into functional groups such as head and packaging, connector, tail, DNA, RNA, nucleotide metabolism, transcription regulation, and hypothetical or unknown function. Genome comparison using gggenomes reveals a modular architecture among the phage genomes in Cluster A (Fig. 4). The modular pattern followed by most of the genomes in this cluster can be described as the following: the left side of the genomes predominantly encodes structural proteins, followed by proteins involved in host lysis, while the right side contains proteins related to nucleotide metabolism, transcription regulation, integration, and excision. Cluster A genomes show high conservation across all of these modules. Notably, most phages in Cluster A encode holin except for phiMMP04, and integrase is present in all phages except for phiCDHM11. The phage genomes in Cluster A exhibit a high ANI value ranging from 60% to 96%. Additionally, the PEQ value among Cluster A members is high as well, ranging from 50% to 90%. Cluster A genomes are divided into three distinct subclusters—A1, A2, and A3 (Fig. S2). Subcluster A1 includes the sole member, pCD5401_3. Phages phiCD506, JD032, JD033, phiCD481-1, phiCDHM11, phiCDHM13, and phiCDHM14 form A2. The A3 subcluster consists of phages CD2301, phiCD08011, and phiMMP04.

#### Cluster B

Cluster B comprises the largest eight genomes, ranging from 113 kbp to 134 kbp, and is characterized by a lower GC content of around 26%. Following their large genome size, these phages encode a substantial number of proteins ranging from 140 to 184 with an 86% coding density. All genomes in Cluster B are yet to be classified and uniquely contain tRNAs. Pangenome analysis revealed the highest number of gene families in this cluster, with 49 core genes (14%) involved in various functions like packaging and structure, DNA, RNA, nucleotide metabolism, transcription regulation, integration and excision, and lysis. A significant portion of the core genes are hypothetical proteins with unknown functions. The genome organization in Cluster B, while containing specific modules like tail, lysis, and integration and excision, does not exhibit any particular pattern. Conservation is relatively low, primarily observed among structural proteins such as portal protein, virion structural protein, and a few tail proteins. Notably, integrase, followed by proteins endolysin and holin that are involved in host lysis, is conserved across the genomes. Synteny breaks are random and inconsistent, indicating sporadic gene transfer events. Diverse ANI values among the genomes in Cluster B are observed, ranging from 32% to 88%. Cluster B contains five subclusters (B1 to B5), three of which (B1, B2, and B3) contain one member each (Fig. S3). Phages LIBA2945 and LIBA6276 form B4, followed by Phages AR1086-1, phiCD211, and phiCDIF1296T forming the B5 subcluster, respectively.

#### Cluster C

Cluster C consists of six genomes with similar sizes ranging from 48 kbp to 53 kbp and an average GC percentage of 29%. These genomes encode between 72 and 88 coding sequences, with a relatively higher coding density. Five members of Cluster C are classified as *Colneyvirus*, while phiCD418 remains unclassified. Five subclusters are identified in Cluster C—C1 to C5, with four (C1, C2, C4, and C5) containing a single member each (Fig. S4). C3 is formed with phages phiCD505 and phiMMP02. Genomic comparison reveals several conserved genes among the cluster 3 genomes. These genomes collectively encode 159 gene families, with 19 core genes (12%), which is the lowest core gene count among the clusters. The core genes in this cluster are associated with functions related to the structural organization of the phage and lysis processes. These genes are confined to the left side of the genomes. Synteny breaks and random insertions-deletions (indels) are observed in the right side of the genomes, which are involved in nucleotide metabolism and host-associated functions.

#### Cluster D

The highly conserved Cluster D contains six genomes classified as *Leicestervirus*. Except for the phage Hain-Saunders-2022a (HS-2022a), the rest of the five phages are around 41 kbp. The genome length of Hain-Saunders-2022a is comparatively smaller, 35 kbp. The genomes have a GC content of around 30%–31% and encode 48–53 proteins with an average 87% coding density. The slight discrepancy observed in genome length in the case of Hain-Sanders-2022a is reflected in the rest of the features as well. The phages phiCD146, phiCD111, CDSH1, AR1075, and phiCD38-2 have high ANI and PEQ values (>83%), forming the subcluster D2 (Fig. S5). In comparison, the ANI value of the sole member of the remaining subcluster D1, phage Hain-Saunders-2022a, is around 45%–50% with the rest of the genome, while the PEQ values are slightly lower, around 35%–37%. It is of important note that two phages, AR1075-1 and phiCD38-2, are almost identical, evidenced by 100% ANI and 99% PEQ values. Cluster D genomes share 64 gene families with 33 (52%) core genes with structural, nucleotide metabolism, transcription regulation, and lysis functions.

#### Clusters E and F

Cluster E represents another highly conserved group, comprising four members classified as *Yongloolinvirus*. Genome sizes within this cluster vary, with phiMMP01 having the smallest genome length at 44 kbp, while the remaining genomes range from 52 kbp to 56 kbp. Three phages within this cluster, namely phiMMP01, phiC2, and phiMMP03, exhibit high ANI and PEQ values, ranging from 71% to 87%. Conversely, CDMH1 displays comparatively lower ANI percentages with the aforementioned phages, averaging around 53%, which is reflected in its relatively lower PEQ values. Consequently, Cluster E is divided into two subclusters with phiMMP01, phiC2, and phiMMP03 forming E2, while the phage CDMH1 is the sole member of the second subcluster, E1 (Fig. S6). In comparison to the other highly conserved cluster D, the core gene percentage (29%) in Cluster E is lower. Notably, among the conserved proteins, structural and hypothetical proteins are prominent.

Among the three members of Cluster F, phiCDHM19 and phiCD119 are classified as *Lubbockvirus*, while CD1801 remains unclassified. Variability in genome length is evident within this cluster, with phiCDHM19 (54 kbp) and phiCD119 (53 kbp) having larger genome sizes compared to CD1801 (44 kbp). Corresponding to the differences in genome sizes, phiCDHM19 and phiCD119 exhibit higher similarity and share high ANI and PEQ values compared to CD1801, resulting in two subclusters with CD1801 forming F1 and phiCD119, phiCDHM19 forming the subcluster, F2 (Fig. S7). The genomes in Cluster F encode a total of 131 gene families, with 39 core genes (30%).

#### Clusters G, H, and I

Clusters G, H, and I each consist of two unclassified members. Members of Cluster H exhibit comparatively smaller genome lengths. Notably, among all clusters, Cluster G displays the lowest coding density. Each cluster demonstrates high conservation, with ANI exceeding 60% and PEQ surpassing 57%. However, Cluster G members have 61.6% ANI between them, resulting in two subclusters: G1 and G2, containing the phages HGP05 and phiSemix9P1, respectively (Fig. S8). Furthermore, these clusters exhibit a high percentage of core genes: Cluster G contains 53 core genes (55%), Cluster H contains 44 core genes (62%), and Cluster I contains 47 core genes (72%). Of particular interest, Cluster I deviates from the typical modular pattern observed in the other clusters. Structural genes are typically located on the left side of the genome, and nucleotide metabolism and integration-related genes are on the right side, a general pattern seen among the other clusters. In Cluster I, however, in the phage phiCDKH01, structural genes are present on the right side of the genome. Genome comparison figures of Clusters B to I are included in the supplementary files (Fig. S10 through S14)

### Phylogenetic analysis and taxonomic inference

**Fig 5:**
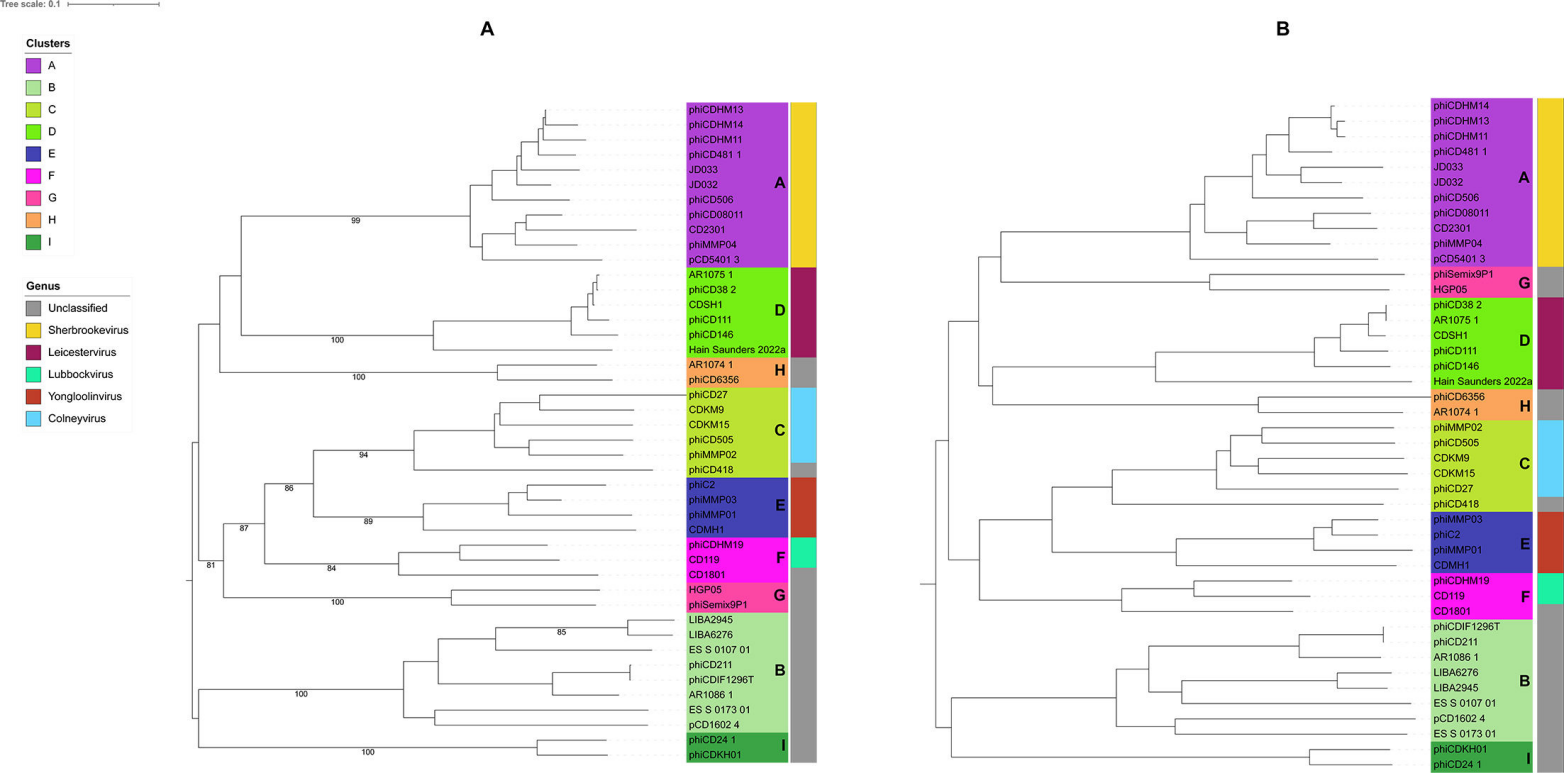
(**A**) Phylogenetic tree based on whole genome generated by VICTOR. Assigned clusters and the currently assigned genus are annotated in different colors as indicated in the legend. Terminal nodes are colored according to clusters, and the colored strip following the nodes represents the genus of the phages. Each cluster is distinguishable as a distinct clade within the tree. (**B**) Proteomic tree generated by ViPTree using only CD phage sequences. Similarly, as the whole-genome-based tree, each cluster forms a distinct clade and is discernible.

Whole-genome-based phylogenetic analysis using VICTOR yielded three trees with three different methods. D0, the recommended method for nucleotide analysis by VICTOR, was selected for further examination. The phylogenomic GBDP tree (Fig. S15) was inferred using the formula D0 yielding an average support of 51%. The numbers above the branches are GBDP pseudo-bootstrap support values from 100 replications. The OPTSIL clustering resulted in 41 species clusters, 5 genus-level clusters, and 1 family-level cluster. However, these suggested taxonomic groupings diverged from the existing CD phage taxonomy. Therefore, the tree was also visualized and annotated in iTOL according to the current taxonomy assignment of the CD phages in the International Committee on Taxonomy of Viruses (ICTV) (Fig. 5A). Furthermore, the proteomic tree generated using ViPTree in query mode, including only the CD phage sequences, was similarly annotated in iTOL (Fig. 5B). Notably, both the genomic and proteomic trees exhibited clustering of phages from the same group. These clusters formed distinct monophyletic clades within the trees with corresponding clusters identifiable in both analyses.

**Fig 6:**
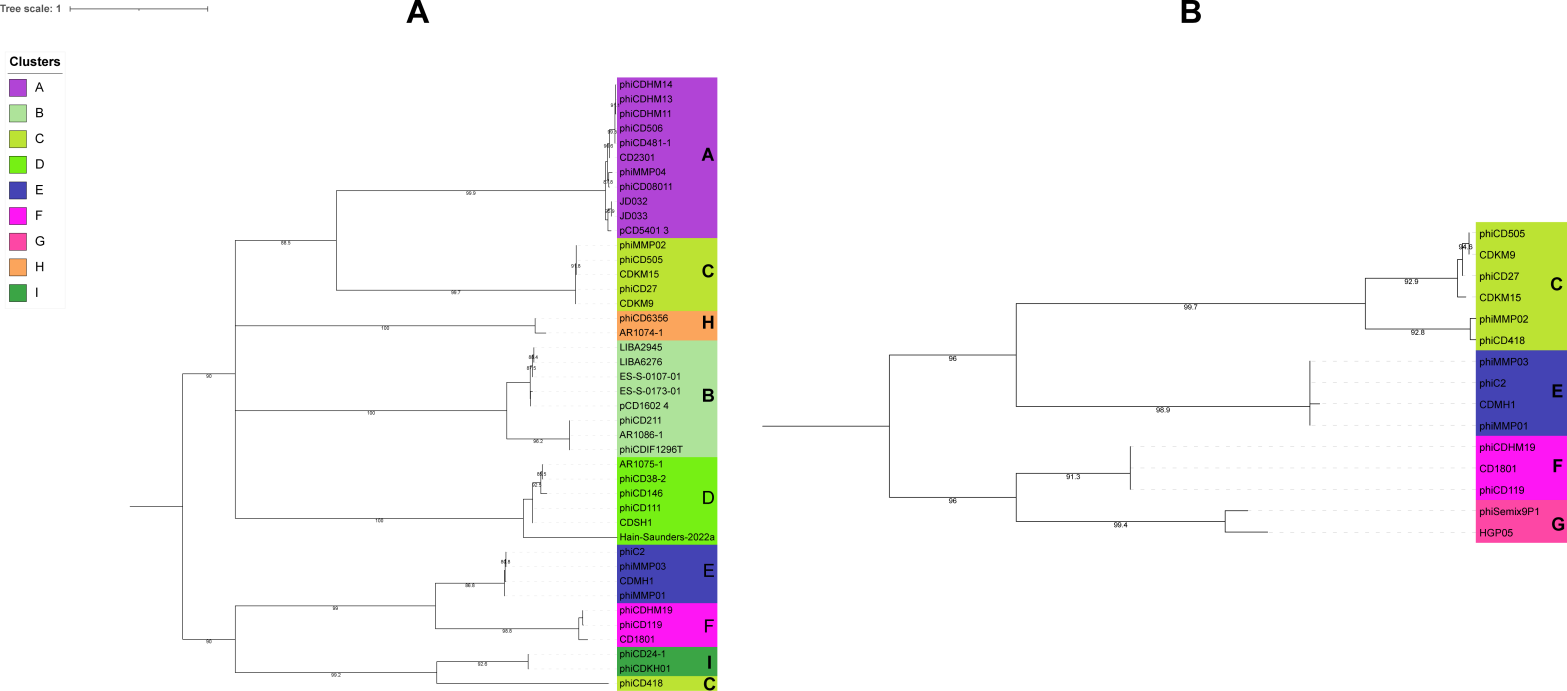
Phylogenetic tree generated with amino acid sequences of (**A**) portal protein gene, (**B**) tail protein XkdN-like gene. The trees were constructed utilizing the ETE3 pipeline implemented on GenomeNet (https://www.genome.jp/tools/ete/). MAFFT was used for alignment followed by alignment trimming using trimAl. The ML tree was inferred using IQ-TREE, best-fit models were LG + F + G4 (**A**), and LG + I (**B**) with branch testing by SH-like aLRT with 1,000 replicates. Terminal nodes are colored according to assigned clusters indicated in the legend. Except for cluster C phage, phiCD418, portal protein genes of other phages form distinct clades with their respective cluster members. Similarly, tail proteins of the phages form separate clades, and each cluster is discernible.

To further evaluate the cluster assignment of phages, we performed single-gene phylogenetic analysis. As discussed earlier (Genomic comparison reveals nine clusters without any singleton), no single gene was consistently present in all the genomes. Therefore, we selected two core genes that are present in multiple clusters: portal protein and tail protein XkdN-like. Portal protein, a core gene for Clusters A, B, D, E, F, H, and I, was used to construct a phylogenetic tree (Fig. 6A). Notably, phages from each aforementioned cluster formed distinct monophyletic clades within the tree. Interestingly, in the case of Cluster C, five out of six members formed a monophyletic clade, while the remaining member phiCD418 clustered with phages from Cluster I. This observation highlights the mosaic nature of these phages. Similarly, the tail protein XkdN-like, a core gene for Clusters C, E, F, and G, was used to generate a phylogenetic tree (Fig. 6B). As observed with the portal protein tree, tail proteins from phages belonging to these clusters formed separate clades. In both trees, each cluster is separately discernible, thus solidifying cluster assignment at the single-gene level.

The current classification system for CD phages recognizes five genera, with 28 classified phages and 16 unclassified. To establish genus-level relationships, the recommended ANI cutoff of 70% by the ICTV was employed. This analysis resulted in the subdivision of cluster members into 21 subclusters, where each subcluster represents a distinct genus. Interestingly, Clusters H and I exhibited 70% ANI, indicating they each represent a single genus on their own. Consequently, this classification approach proposes the revision of CD phages into a total of 23 genera.

Furthermore, we observed that each existing genus aligns with a distinct cluster. Notably, all members within a cluster share >30% nucleotide identity and possess at least 19 core genes. This high degree of sequence similarity and shared core genes suggests a subfamily-level relationship among each subcluster (genus). This subfamily classification is further supported by the single gene phylogeny analysis discussed previously. Based on these findings, we propose elevating the existing genera to the subfamily level and establishing 23 novel genera for CD phages.

**Fig 7:**
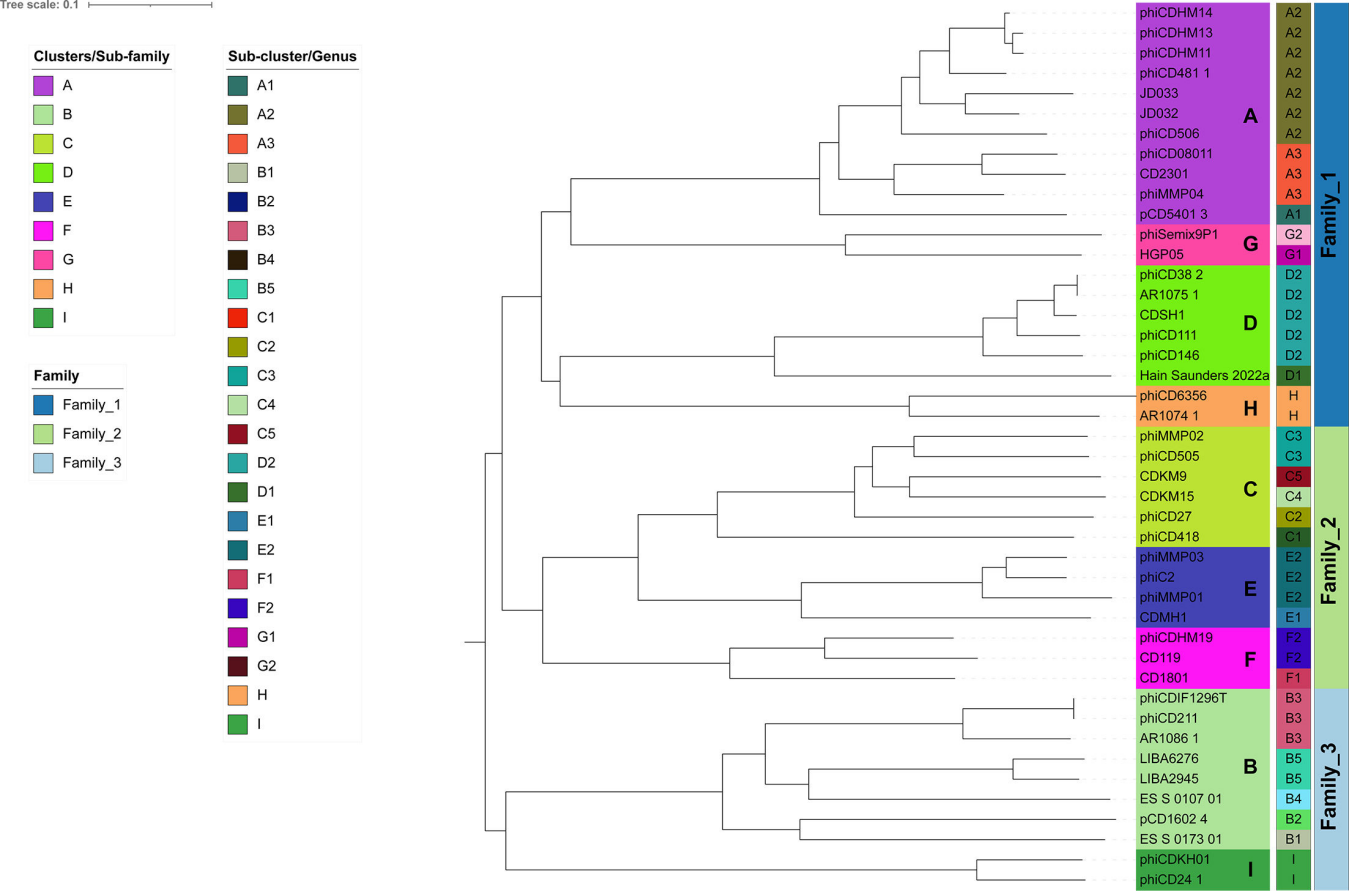
Proteomic tree annotated with updated taxonomic information. Terminal nodes are colored according to cluster/subfamily assignment. The following color strip represents subcluster/genus. Each subcluster/genus is labeled in the color strip as well. The final color strip represents family-level relationships among the clusters.

To investigate family-level relationships, the tree generated in reference mode considers only related (SG > 0.02) viral sequences (Fig. S16). However, the lack of a significant number of shared orthologous genes, a requirement by ICTV for a single family, necessitated further analysis. We explored inter-cluster gene sharing and identified three monophyletic groups based on the recommended ICTV criteria. The first group (Clusters A, D, G, and H) shared four core genes, the second group (Clusters C, E, and F) shared eight core genes, and the third group (Clusters B and I) shared seven core genes. Based on this analysis, we propose classifying CD phages into three families: the first comprising Clusters A, D, G, and H, the second comprising Clusters C, E, and F, and the third comprising Clusters B and I (Table 2). The revised proteomic tree depicts the genus and family-level relationships among the corresponding clusters and cluster members (Fig. 7).

**TABLE 2:** Cluster and taxonomic assignment of *C. difficile* phages

Family	Cluster/subfamily	Number of gene families (and core genes)	Subcluster/genus	Phages
Family_1	Cluster A	85 (29)	A1	pCD5401_3
			A2	phiCD506
				JD032
				JD033
				phiCD481-1
				phiCDHM11
				phiCDHM13
				phiCDHM14
			A3	CD2301
				phiCD08011
				phiMMP04
	Cluster D	64 (33)	D1	Hain-Saunders-2022a
			D2	CDSH1
				phiCD38-2
				phiCD111
				phiCD146
				AR1075-1
	Cluster G	96 (53)	G1	HGP05
			G2	phiSemix9P1
	Cluster H	71 (44)	H	phiCD6356
				AR1074-1
Family_2	Cluster C	159 (19)	C1	phiCD418
			C2	phiCD27
			C3	phiCD505
				phiMMP02
			C4	CDKM15
			C5	CDKM9
	Cluster E	139 (40)	E1	CDMH1
			E2	phiC2
				phiMMP01
				phiMMP03
	Cluster F	131 (39)	F1	CD1801
			F2	phiCD119
				phiCDHM19
Family_3	Cluster B	345 (49)	B1	ES-S-0173-01
			B2	pCD1602_4
			B3	AR1086-1
				phiCD211
				phiCDIF1296T
			B4	ES-S-0107-01
			B5	LIBA2945
				LIBA6276
	Cluster I	65 (47)	I	phiCD24-1
				phiCDKH01

### Diversity of the lytic proteins

**Fig 8:**
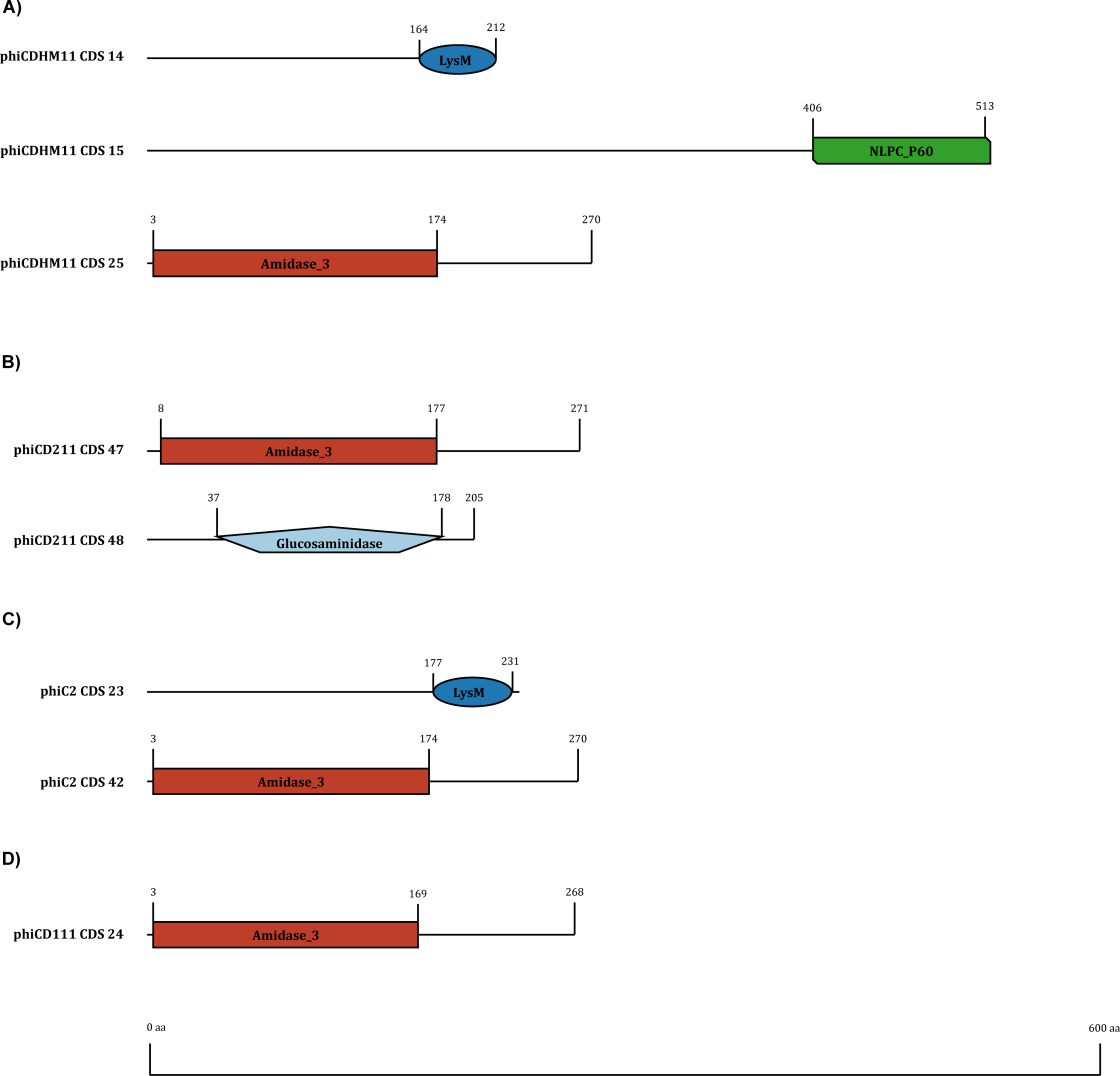
Endolysin organization and domain diversity in CD phage genomes. (**A**) Three endolysins encoded in a single genome. Two are encoded consecutively, while the third endolysin is separately encoded. (**B**) Two endolysins encoded consecutively. (**C**) Two endolysins encoded separately. (**D**) Single endolysin encoded in a genome. Representative endolysins with their corresponding coding sequence numbers are included to indicate their position in the genome.

**Fig 9:**
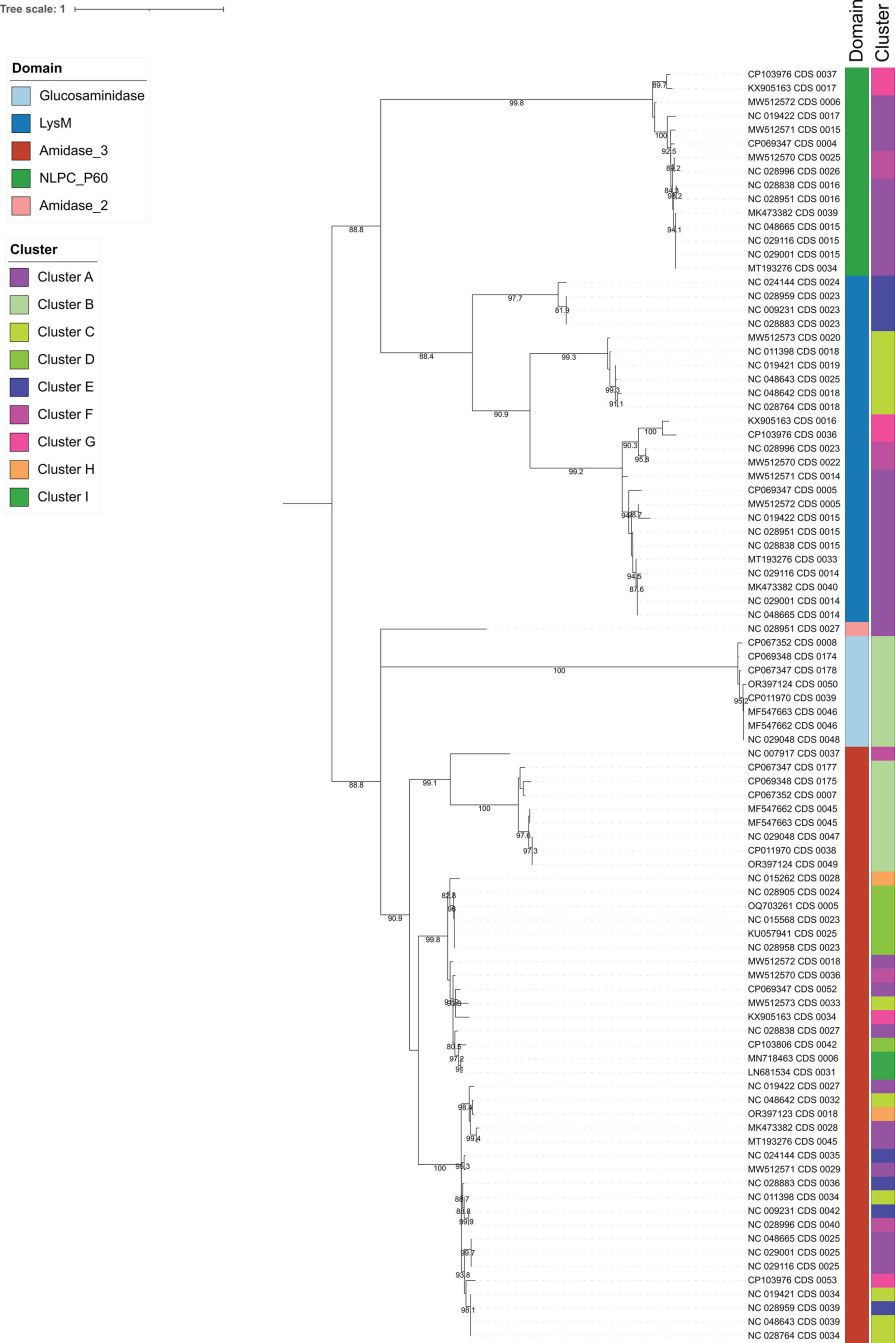
Phylogenetic tree of endolysin sequences of CD phages. The tree was constructed utilizing the ETE3 pipeline implemented on GenomeNet (https://www.genome.jp/tools/ete/). MAFFT was used for alignment followed by alignment trimming using trimAl. The ML tree was inferred using IQ-TREE, best-fit model was LG + F + G4 with branch testing by SH-like aLRT with 1,000 replicates. The color strip following the terminal nodes represents domains. The second color strip represents the cluster of the corresponding host phage. Endolysins from the same domain form distinct clades, except the single amidase_2-containing endolysin. Clusters, however, are widely dispersed since it was observed that several single phage encodes multiple endolysins.

**Fig 10:**
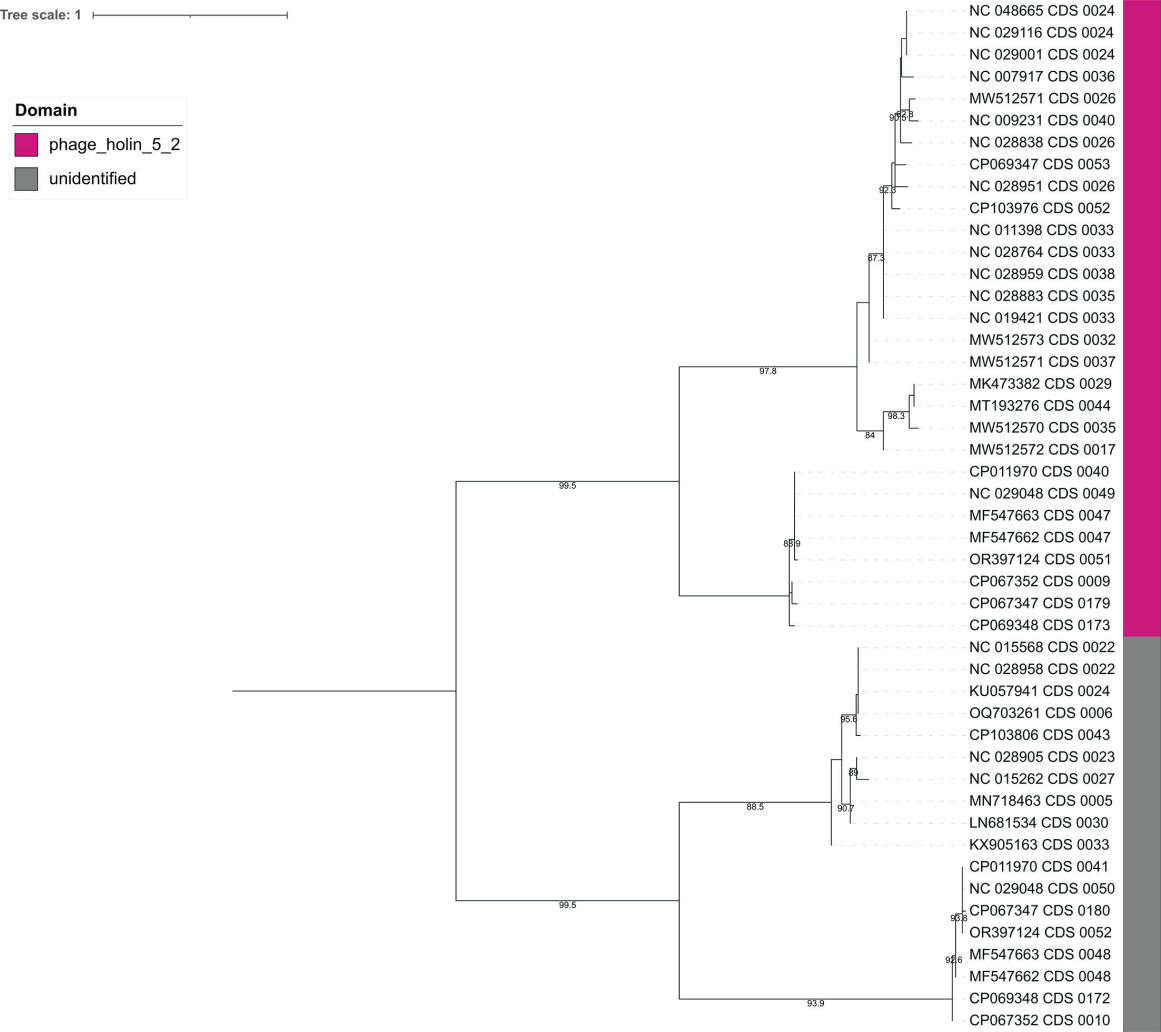
Phylogenetic tree of holin sequences of CD phages. The tree was constructed utilizing the ETE3 pipeline implemented on GenomeNet (https://www.genome.jp/tools/ete/). MAFFT was used for alignment followed by alignment trimming using trimAl. The ML tree was inferred using IQ-TREE, best-fit model was JTT + R2 with branch testing by SH-like aLRT with 1,000 replicates. Holins form two major clades. One consists of phage_holin_5_2-containing holins, and the other clade contains holins of unidentified domains.

The lysis module of CD phages consists of two proteins: endolysins and holins. In this study, we identified 92 endolysins and 47 holins. Endolysins were clustered into five phams—pham_1 (*n* = 43), pham_12 (*n* = 25), pham_32 (*n* = 15), pham_108 (*n* = 8), and pham_667 (*n* = 1). Endolysin sequences within a specific pham exhibit identical domain architecture. Pham_1, pham_32, pham_108, and pham_667 contain catalytic domains amidase_3 (PF01520), NLPC-P60 (PF00877), glucosaminidase (PF01832), and amidase_2 (PF01510), respectively, while endolysins in pham_12 possess the cell wall binding LysM domain (PF01476). CD phages demonstrate four distinct endolysin organizations: (i) three endolysins with two encoded consecutively, while the third endolysin is encoded separately, (ii) two endolysins encoded consecutively, (iii) two endolysins encoded separately, and (iv) a single endolysin encoded in a genome (Fig. 8). Phages in Clusters A, F, and G encode three types of endolysins containing amidase_3/amidase_2, NLPC_P60, and LysM domains, with the NLPC_P60 and LysM domain-containing endolysins being encoded by two consecutive genes. A similar arrangement is observed in Cluster B phage-derived endolysins, where two types of endolysins containing the catalytic domains glucosaminidase and amidase_3 are encoded by consecutive genes. Phages in Clusters C and E also encode two types of endolysins, with one containing the catalytic amidase_3 domain and the other containing the cell wall binding LysM domain, although they are not encoded consecutively. Conversely, phages in Clusters D, H, and I encode only one type of endolysin containing the catalytic amidase_3 domain. Endolysins of the same domain and pham form distinct clades in the phylogeny (Fig. 9) except for amidase_2-containing endolysin from the orpham, pham_667, which is a singleton encoded by the CD phage phiCD481-1. Additionally, CD phage holins were clustered into three protein families: pham_5, pham_63, and pham_91. Pham_5 holins contain phage_holin_5_2 domain (PF16079), while the rest of the holin families did not yield any conserved domain when searched in phmmer. Similar to endolysins, three holin families form three distinct clades (Fig. 10).

**Fig 11:**
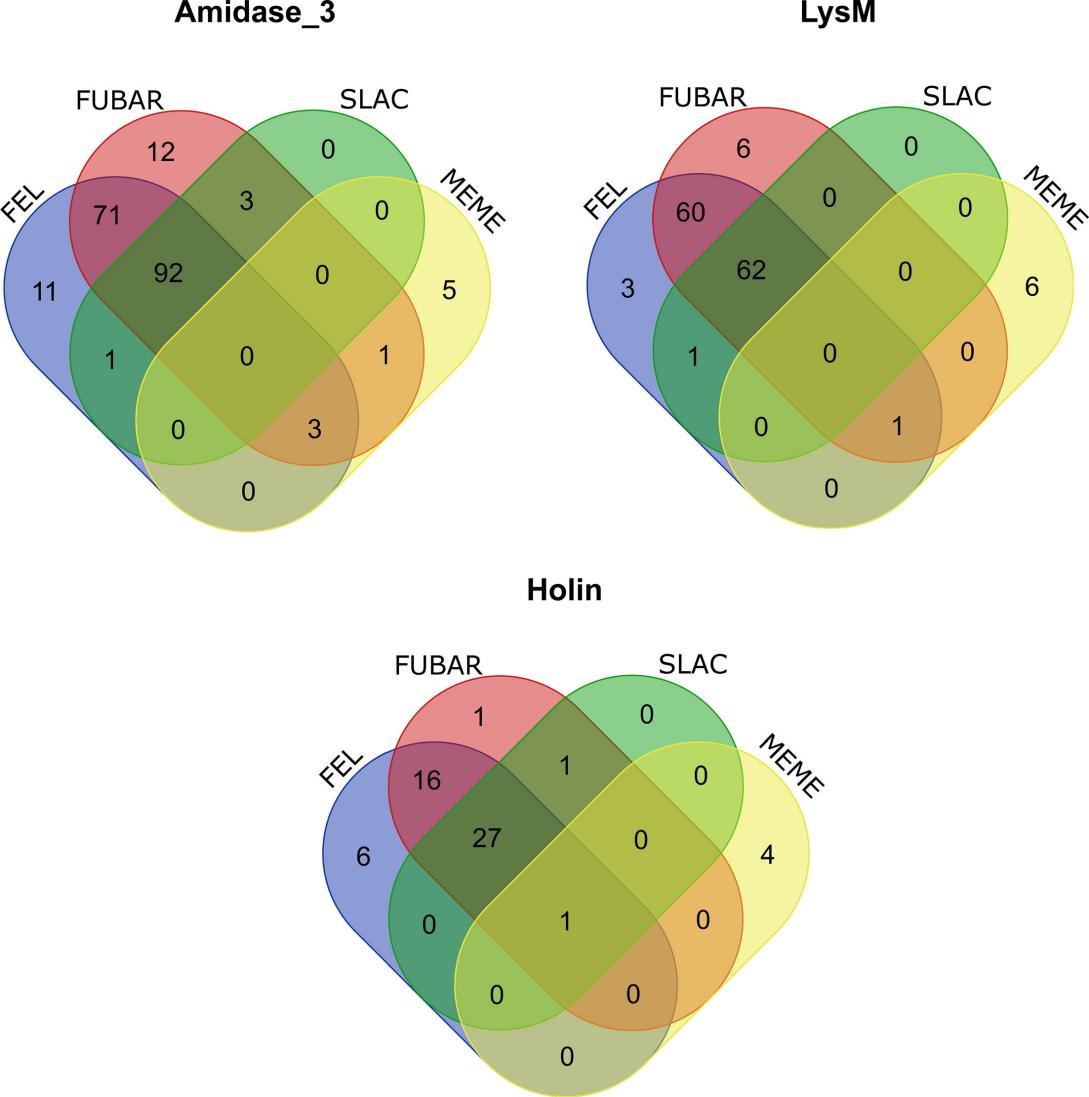
Comparison of selection sites predicted by various models in the case of amidase_3, LysM domain-containing endolysin genes, and phage_holin_5_2 domain-containing holin genes. Based on the common sites predicted by all four models FEL, FUBAR, SLAC, and MEME, negative selection is prevalent in the endolysin and holin genes.

Notably, most of the phages encode highly conserved amidase_3-containing endolysins (Fig. S17) with 25 out of 44 phages also encoding highly conserved LysM domain-containing endolysins. This conservation is reflected in the selection pressure acting on these two endolysins. Results from Datamonkey show strong evidence of purifying selection. Only the MEME result was suggestive of episodic diversifying selection at 9 of 296 sites and 7 of 211 sites for amidase_3-containing and LysM-containing endolysins, respectively. On the contrary, FUBAR suggests 182 of 296 sites, and 129 of 211 sites under pervasive purifying selection for amidase_3 and LysM, respectively. In the case of holins, selection pressure analysis was conducted only for pham_5 holins due to the absence of identified domains and a low number of proteins (<10) in the other phams after duplication removal. Holins, particularly those in phage_5, are subjected to strong purifying selection, as evidenced by the results of Datamonkey. Furthermore, the comparison of sites under selective pressure detected in each model is represented by Venn diagrams, which illustrate the prevalence of purifying selection in CDP endolysins (Fig. 11). In the case of amidase_3-containing endolysins, 92 sites under negative/purifying selection were shared by the FEL, FUBAR, and SLAC models. Five unique positive sites were detected by MEME, while three sites were shared with SLAC and FUBAR, and one site was unique to FUBAR. Similarly, for LysM-containing endolysins, 62 negative sites were detected in FEL, FUBAR, and SLAC. MEME shared one common site with FEL and FUBAR, while 27 sites were common in FEL, FUBAR, and SLAC. Table S2 summarizes the results from Datamonkey.

### Expanded diversity of *Clostridioides difficile* phages across uncultivated viral genomes

A total of 2,790 CD phage genomes were identified in the IMG/VR v4 data set using “clostridioides” as a search keyword. From this pool, we selected 297 high-quality genomes, excluding RefSeq genomes, for further analysis. To compare these uncultivated CD phages with sequenced genomes, we chose nine representative genomes, one from each previously defined cluster, for proteome-based clustering using PhamClust. Out of the 297 genomes analyzed, 285 aligned with the representative genomes, fitting into the nine predefined clusters (Table S3). BLASTn results showed high similarity (>70% nucleotide identity) between the representative genomes and other genomes within each cluster. Additionally, five genomes formed two new clusters, designated Clusters J and K, while seven genomes were identified as singletons. According to vContact2 results, all *C. difficile* phages grouped into distinct viral clusters (VCs), while the singletons identified in PhamClust remained as outliers. Consistent with prior findings, CD phages exhibited minimal similarity to other phages, as supported by the VCs (Table S3) and network analysis (Fig. S18). However, some deviations were observed. For instance, three genomes (IMGVR_UViG_2671180842_000004, IMGVR_UViG_2848092901_000003, and IMGVR_UViG_2859501880_000003) clustered with *Streptococcus* phage Javan630 (MK448997), while the singleton IMGVR_UViG_2938479101_000001 clustered with phages known to infect gram-negative hosts. Interestingly, we identified two lytic phages, IMGVR_UViG_2938479101_000001 and IMGVR_UviG_2541047262_000002, based on consensus results from BacPhlip and PhaTyp, both classified as singletons. Notably, IMGVR_UviG_2541047262_000002, the smallest genome among all identified phages, belongs to the *Microviridae* family. To confirm, a manual BLASTn search across *Microviridae* genomes in the ICTV database revealed ~99.91% similarity to *Escherichia coli* phage phiX174 (J02482.1).

## DISCUSSION

*Clostridioides difficile* is a major healthcare concern. However, therapeutic options, particularly for recurrent infections, remain limited. Phages and phage-derived protein endolysins offer a promising alternative due to their high target specificity. Despite this potential, our understanding of *C. difficile* phages at the genomic level is limited. This study addresses this gap by performing a comprehensive comparative genomic analysis of all available sequenced *C. difficile* phages, revealing significant insights into their diversity and taxonomy.

We observed a diverse range in genome lengths, with the number of coding sequences (CDS) corresponding proportionally to genome size. This observation aligns with established trends in phage biology. Despite this variability in genome length, CD phage genomes exhibited a low GC content within a narrow range (26%–31%), similar to that of their host (29, 63). We found that larger genome sizes were associated with the presence of tRNAs, aligning with previous findings (64). However, this correlation was not observed in phages infecting *Pseudomonas* or *V. cholerae*, where genome length did not influence tRNA presence (25, 27). Computational searches for virulence genes identified a single result, the phage phiSemix9P1, which notably is the first CD phage reported to contain a binary toxin gene (65).

The clustering analysis identified nine distinct CD phage clusters, with no singletons, which is unexpected given the considerable number of singletons in other phage studies (21, 25–27, 66, 67). This absence of singletons might change as more CD phages are sequenced. Previous studies utilized whole-genome dot plots and nucleotide identity for clustering (22, 25–28, 68, 69); however, we emphasized protein sharing for a more accurate depiction of phage relationships, consistent with approaches used in *Gordonia* and *Staphylococcus* phages (24, 70). Using a 35% amino acid identity and 80% coverage threshold as in other studies (23–26, 28, 70), we clustered the proteins into phams. Based on the shared phams, the genomes were grouped into clusters. These clusters were supported by similar genome architecture, core gene presence, GC content, CDS number, and nucleotide similarity. Phylogenetic analyses further validated these clusters, which formed well-defined monophyletic clades. Despite less stringent criteria, no core gene was universally identified, necessitating the identification of core genes across multiple clusters for single-gene phylogeny.

The existing taxonomic classification of CD phages does not adhere to the recommended criteria for genus demarcation. By applying the ICTV-recommended 70% nucleotide identity criterion for genus demarcation (62), we propose a revision of the current genus assignments (30). Our findings suggest that the current genera should be elevated to subfamilies, reassigning corresponding phages into 23 new genera. This mirrors the taxonomic structuring observed in *Acinetobacter* phage genomes, which grouped 139 phages into 8 subfamily clusters and 46 genus-level subclusters (23). It is to be noted that Clusters H and I, each with only two members and over 70% ANI between them, currently represent individual genera. However, since clusters can be interpreted as subfamilies, future discoveries of more phages in these clusters may lead to the creation of multiple genera, clarifying their subfamily-level relationships. For family-level relationships, our proteomic tree analysis indicated that CD phages form a distinct clade, separate from other phages, suggesting the need for a new family designation. This conclusion is supported by the VICTOR-assigned taxonomy (Fig. S15). However, the absence of core genes or orthologs among these phages complicates the family-level assignment, a criterion emphasized by ICTV (30). We therefore explored inter-cluster relationships, identifying multiple clusters that share significant core genes, potentially representing a single family based on ICTV recommendations. Similar criteria were used to classify Lak Megaphages and Epsilon CrAss-like phages recently (71, 72).

Previous studies and our computational predictions describe CD phages as temperate (6). This poses challenges for phage therapy due to isolation difficulties and the potential for reversion to lysogeny (16, 29). Phage-encoded lytic proteins, particularly endolysins, emerge as promising alternatives for therapeutic applications (19). Our analysis of the lytic modules in CD phages revealed a diverse endolysin organization, with distinct domain arrangements. Previous studies on *Staphylococcus* phages identified four types of endolysin organization: single gene, two spliced genes, two adjacent genes, and a single gene with a secondary translational site (24). In CD phages, we observed single-gene and two adjacent-gene organizations, with separate encoding of catalytic and cell wall binding domains. Interestingly, in three CD clusters (A, F, and G), we found that two consecutive genes encode catalytic and cell wall binding domains separately, while an additional distant catalytic domain is also encoded. This suggests the possibility of these genes working together in cell wall cleavage (24). Further analysis is required to determine if the three domains function in conjunction. The catalytic amidase_3 domain and the cell wall binding LysM domain are highly conserved across CD phages, indicative of strong purifying selection, as supported by the Datamonkey analysis.

A key limitation in our study is the small genome pool, which may not fully reflect the diversity of CD phage populations across various environments. Moreover, our stringent criteria for taxonomic relationships have introduced numerous genera and multiple families within a relatively small data set. To address this limitation, we compared sequenced CD phage genomes with those in the IMG/VR v4 data set, one of the largest collections of uncultivated viral genomes. Out of 297 genomes, 285 were clustered within our previously defined clusters, suggesting substantial gene sharing among CD phages. Results from vContact2 further confirmed the uniqueness of CD phages, suggesting a need for new families and genera specific to these phages. Furthermore, the BLASTn results indicated that each representative phage exhibited >70% similarity with its cluster members. This aligns with the nucleotide similarity threshold recommended by the ICTV for genus assignment, which we also applied to the sequenced phages. This suggests that, despite the larger data set, CD phages should still fit within our defined genera and families, reinforcing the validity of our analysis. Notably, the presence of virulent phages and others with similarity to phages infecting gram-negative hosts among singletons hints at an underexplored diversity within the CD phage population. Future studies incorporating more extensive data sets may further uncover this diversity, potentially yielding new insights into the biology and therapeutic potential of CD phages.

### Conclusion

This study provides a comprehensive analysis of *C. difficile* phages, revealing their diverse characteristics, taxonomic landscape, and potential for therapeutic applications. The study identified nine distinct clusters of CD phages with varying genome sizes, GC content, and gene repertoires. Based on average nucleotide identity, protein sharing, and core gene content, the study proposes a revised taxonomic classification, elevating current genera to subfamilies and establishing 23 new genera for CD phages. Furthermore, the analysis of the lytic module suggests the presence of diverse endolysin organizations and strong purifying selection acting on catalytic and cell wall binding domains. These findings provide valuable insights into the biology of *C. difficile* phages and their potential as therapeutic agents against *C. difficile* infections.

## SUPPLEMENTAL MATERIAL

Supplemental table legends spectrum.01431-25-s0001.docx Legends for Tables S1 to S3.

Supplemental figures spectrum.01431-25-s0002.pdf Figures S1 to S18.

Table S1 spectrum.01431-25-s0003.xlsx Cluster-wise PEQ values among the cluster members.

Table S2 spectrum.01431-25-s0004.xlsx Summary of Datamonkey results. Sites under selection inferred from various programs along with common sites detected in the programs are included in the table.

Table S3 spectrum.01431-25-s0005.xlsx Blast matches.

ASM does not own the copyrights to Supplemental Material that may be linked to, or accessed through, an article. The authors have granted ASM a non-exclusive, world-wide license to publish the Supplemental Material files. Please contact the corresponding author directly for reuse.
