# Supplementary material for: Comparative genomics reveals diversity and taxonomic relationships among Clostridioides difficile phages
Source: Microbiol Spectr. 2025 Nov 12;13(12):e01431-25. doi: 10.1128/spectrum.01431-25 (PMC12671070; doi:10.1128/spectrum.01431-25)
Supplement: Supplemental table legends — Legends for Tables S1 to S3. [file spectrum.01431-25-s0001.docx]

**Supplementary table legends:**

**Table S1:** Cluster-wise PEQ values among the cluster members.

**Table S2:** Summary of Datamonkey results. Sites under selection inferred from various programs along with common sites detected in the programs are included in the table.

**Table S3:** Table S3 presents data from the comparison of the sequenced *C. difficile* (CD) phages with the IMG/VR v4 dataset. The table includes the following information: metadata for all high-quality genomes of uncultivated CD phages from the dataset; BLAST results of the *Microviridae* CD genome against genomes of the entire *Microviridae* family; cluster-wise PEQ scores (from Cluster A to Cluster K); list representing the singleton genomes; overview of viral clusters from vContact2; and BLAST results of the representative genomes against the complete IMG/VR v4 dataset.
