# Supplementary material for: Comparative genomics reveals diversity and taxonomic relationships among Clostridioides difficile phages
Source: Microbiol Spectr. 2025 Nov 12;13(12):e01431-25. doi: 10.1128/spectrum.01431-25 (PMC12671070; doi:10.1128/spectrum.01431-25)
Supplement: Supplemental figures — Figures S1 to S18. [file spectrum.01431-25-s0002.pdf]

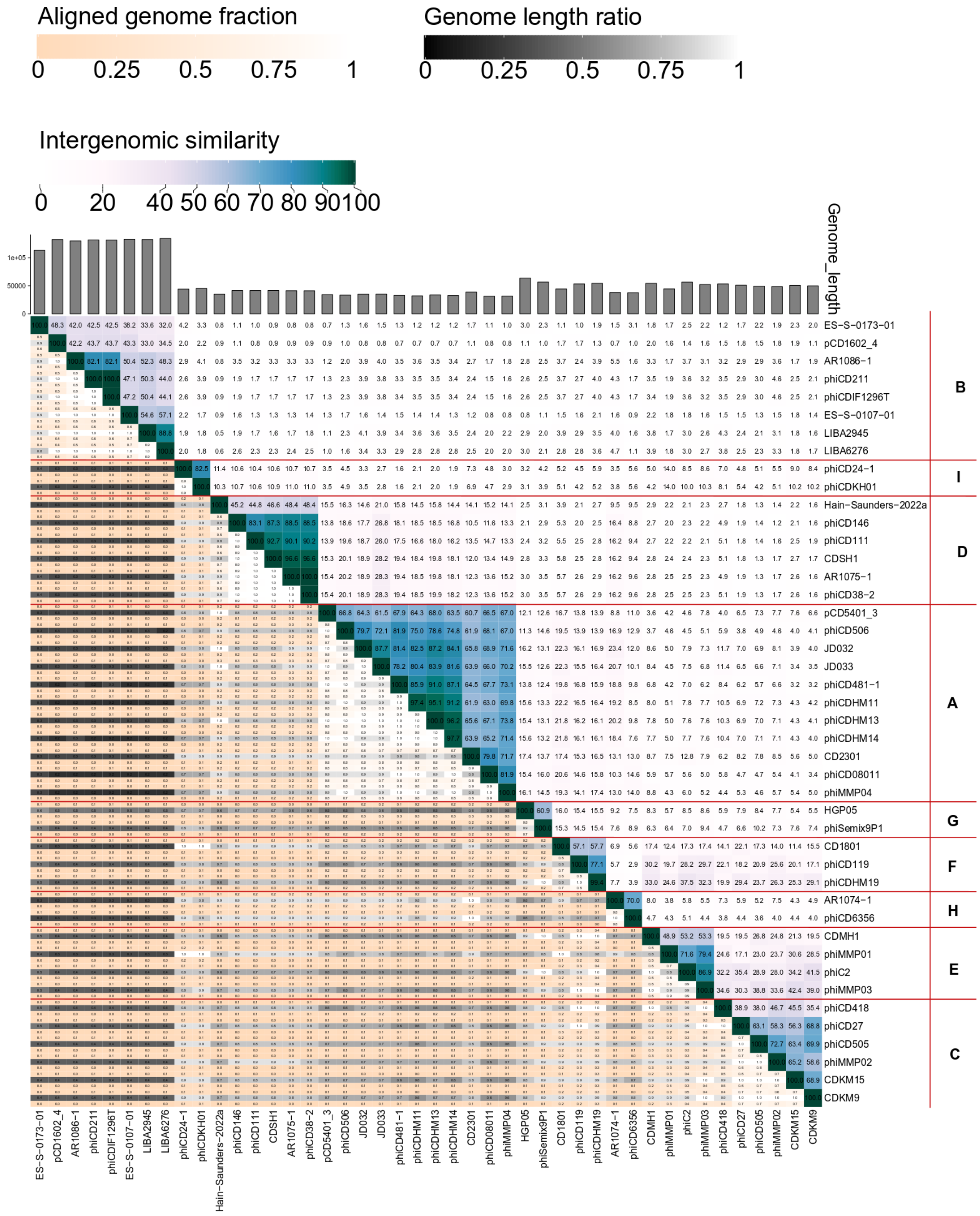

Figure S1: Heatmap of ANI values among the CD phage genomes. Assigned clusters are shown on the right vertical axis. Clusters were defined by high ANI values.

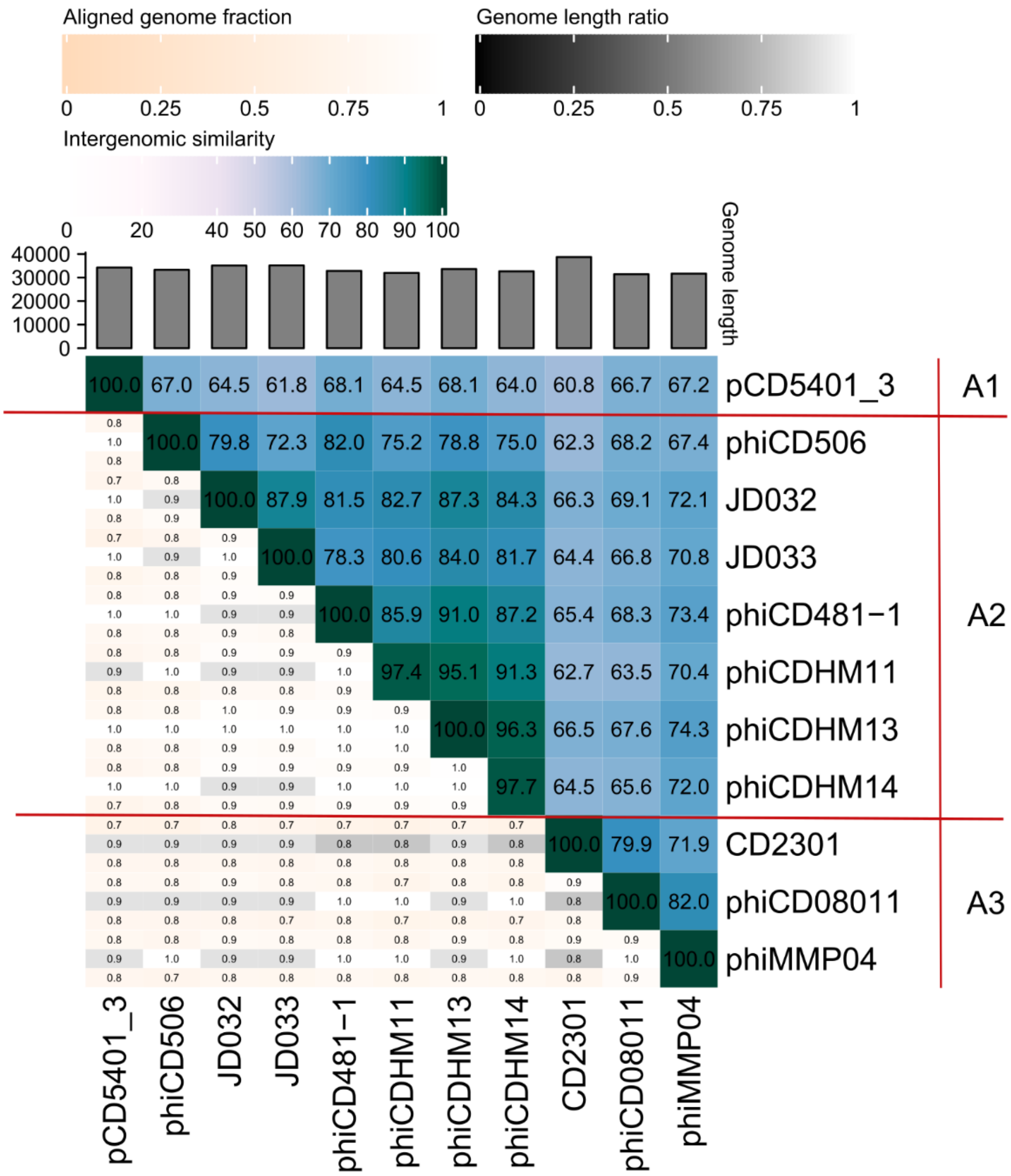

Figure S2: Heatmap of ANI values among the members of cluster A with the subclusters highlighted.

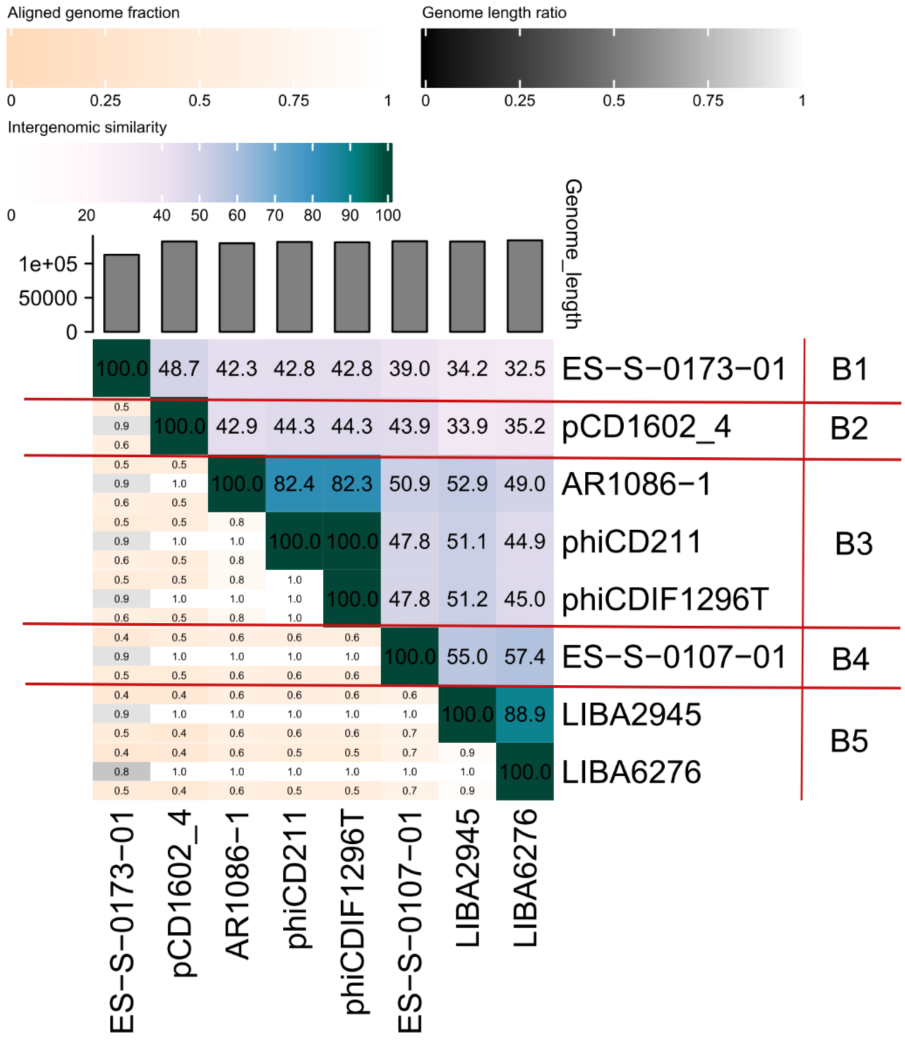

Figure S3: Heatmap of ANI values among the members of cluster B with the subclusters highlighted.

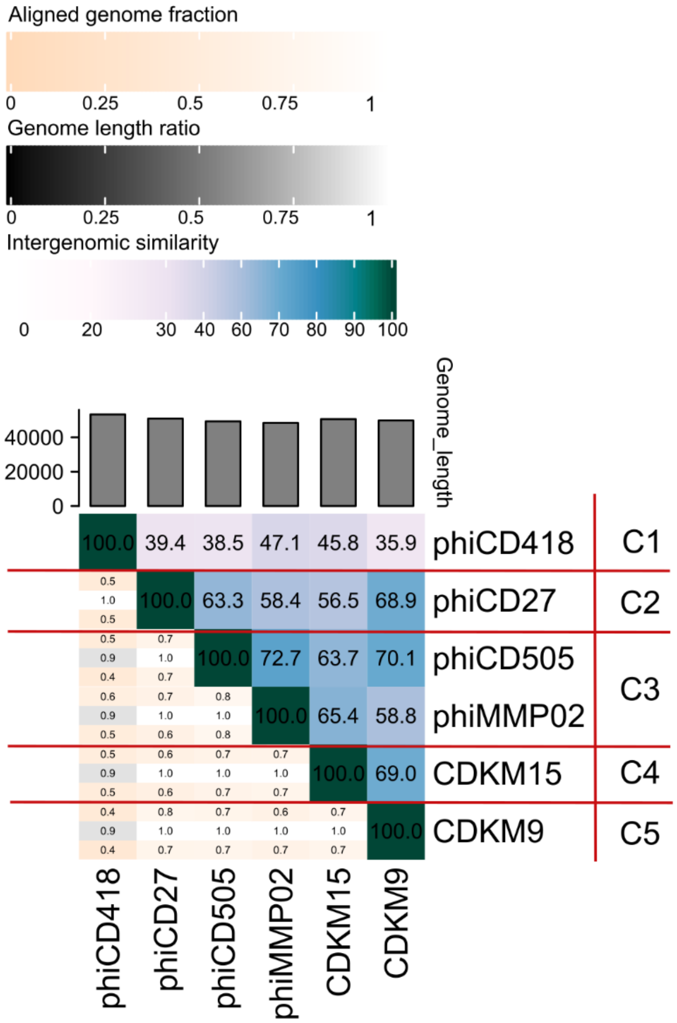

Figure S4: Heatmap of ANI values among the members of cluster C with the subclusters highlighted.

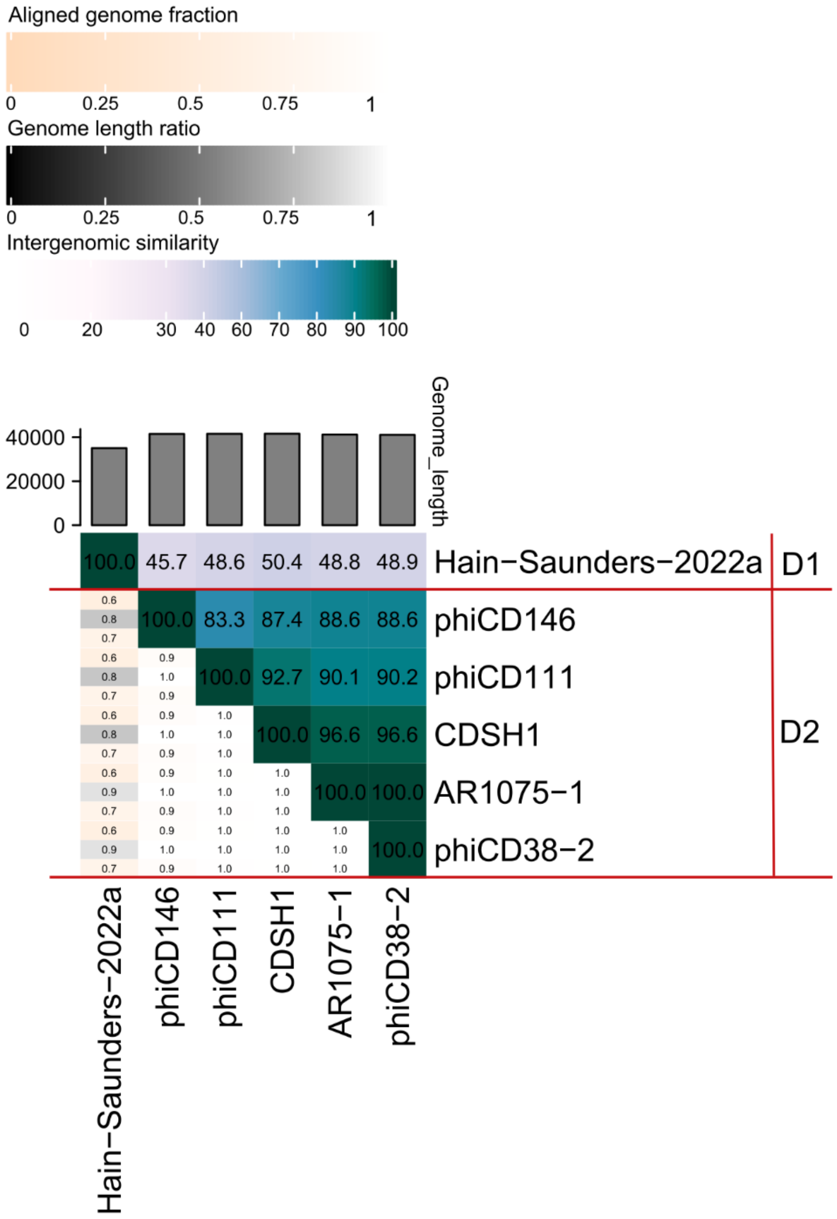

Figure S5: Heatmap of ANI values among the members of cluster D with the subclusters highlighted.

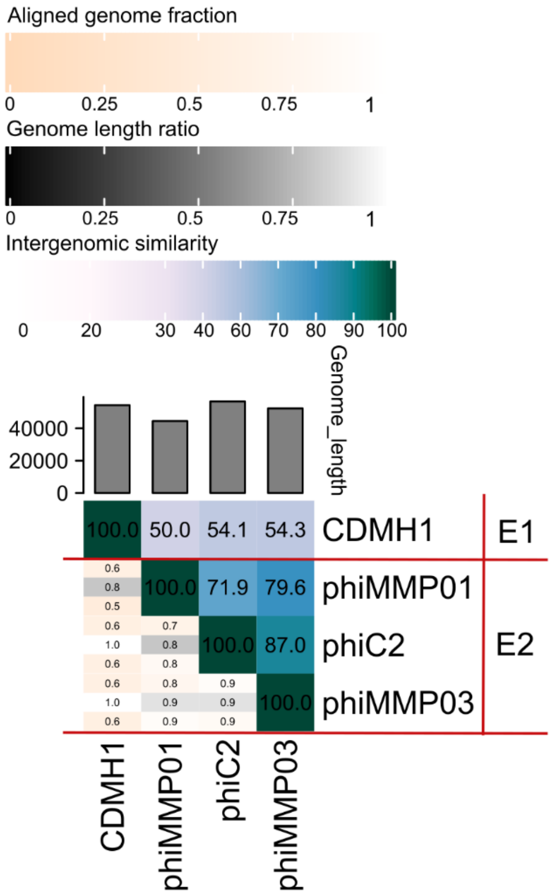

Figure S6: Heatmap of ANI values among the members of cluster E with the subclusters highlighted.

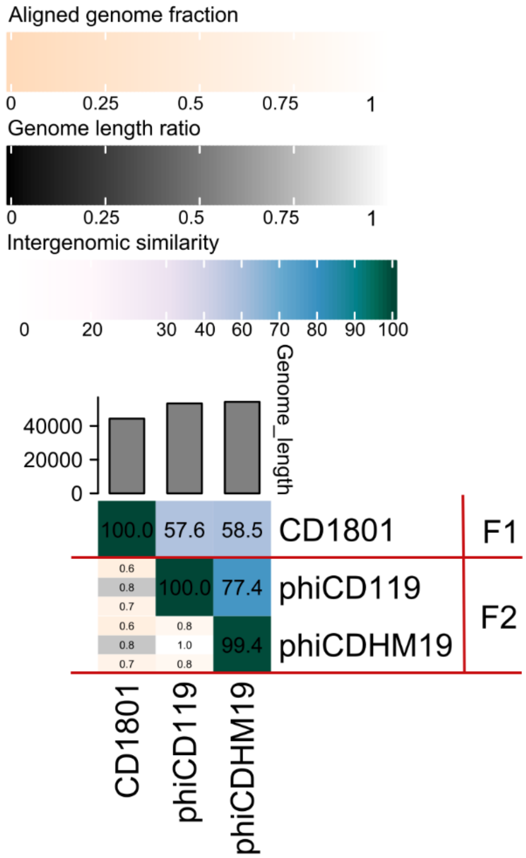

Figure S7: Heatmap of ANI values among the members of cluster F with the subclusters highlighted.

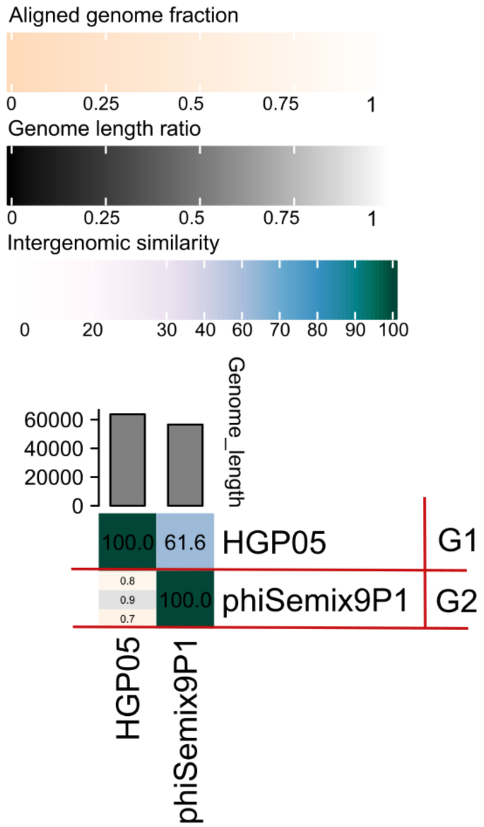

Figure S8: Heatmap of ANI values among the members of cluster G with the subclusters highlighted.

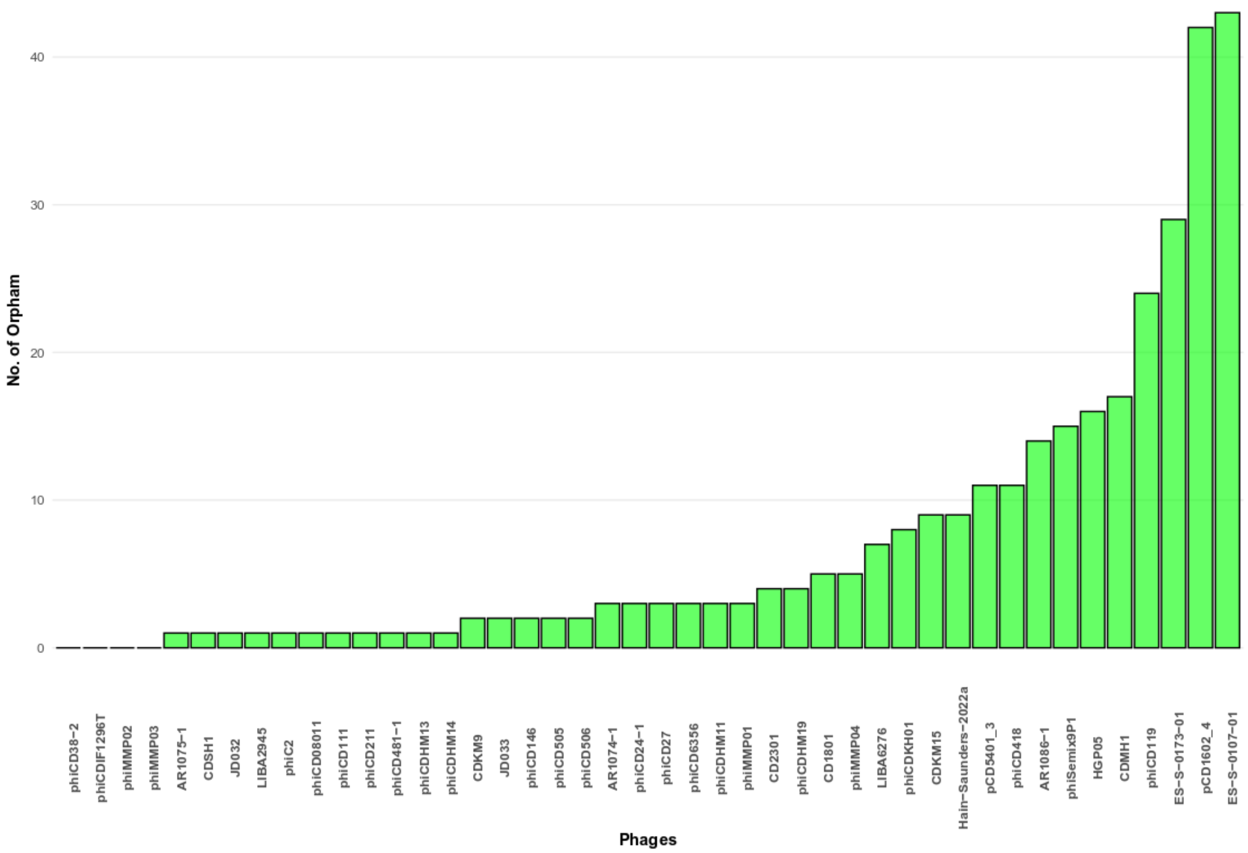

Figure S9: Bar plot depicting the number of orphams per phage. Phages (X-axis) are ordered in ascending order based on the number of orphams on the Y-axis.

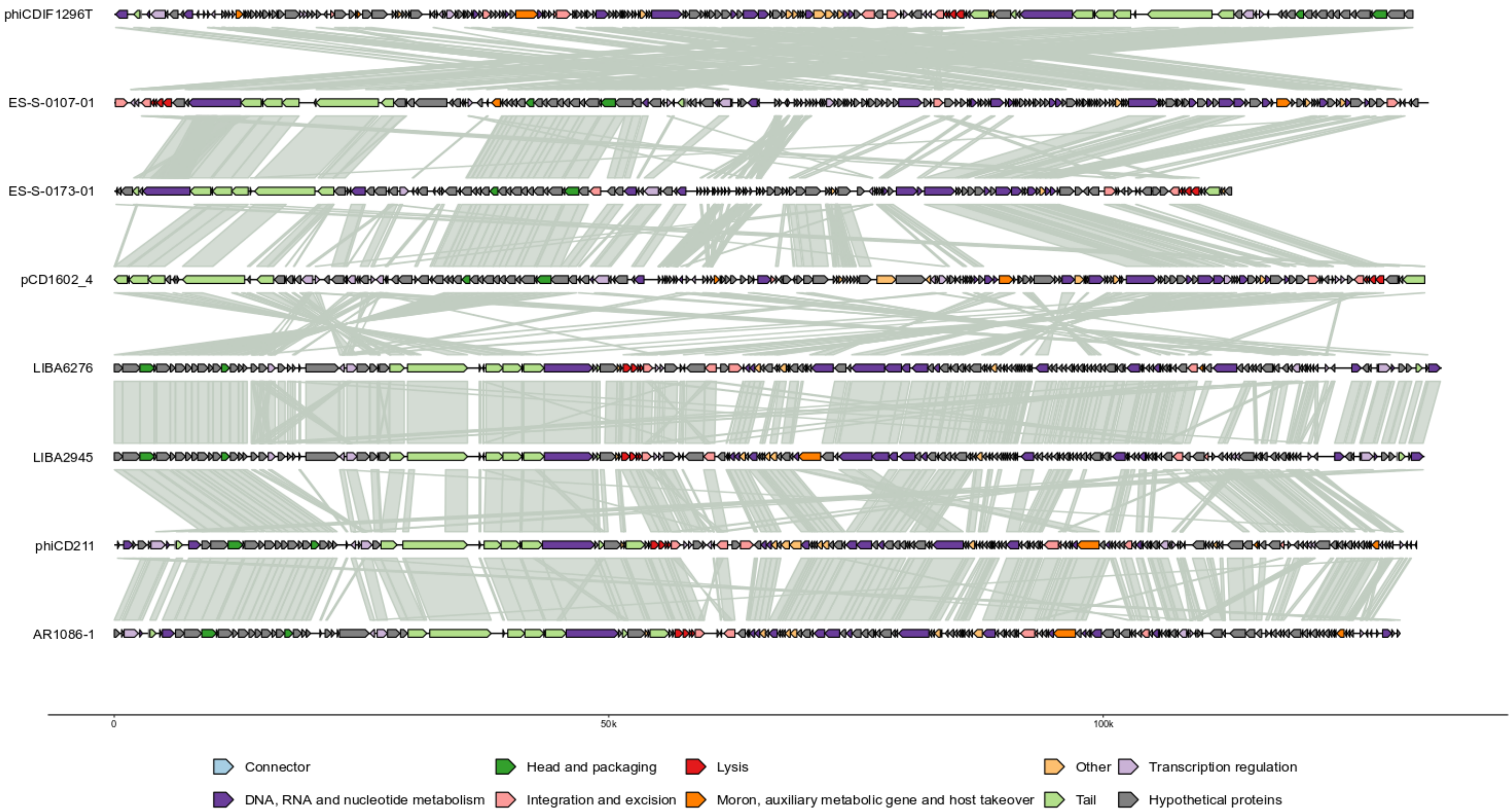

Figure S10: Genome comparison among cluster B phage genomes. The large genomes of cluster B showed the least amount of genomic conservation among the clusters.

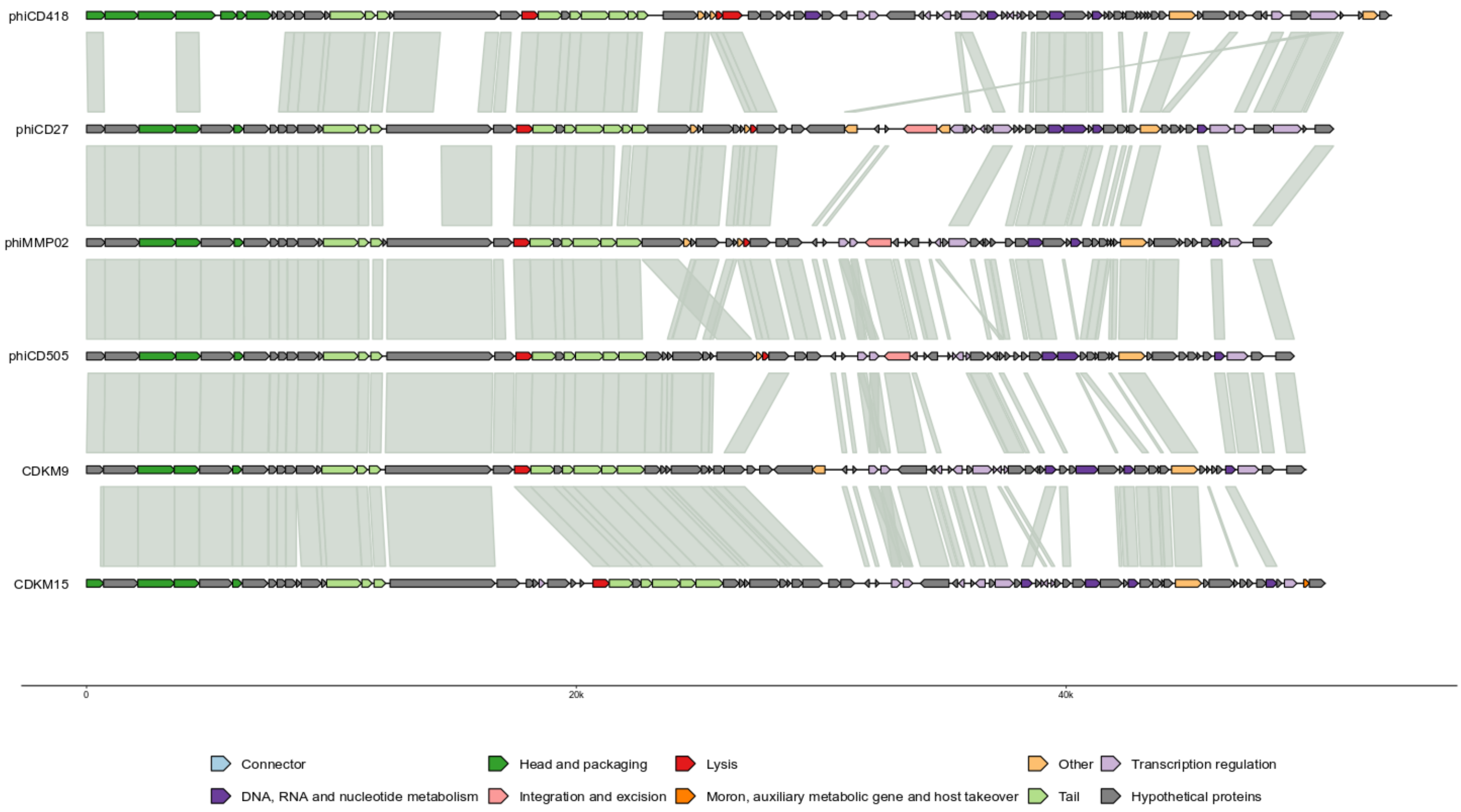

Figure S11: Genome comparison among cluster C phage genomes. Despite being in the same cluster, the phage phiCD418 has relatively low similarity compared to the other five genomes, which is also observed in the figure.

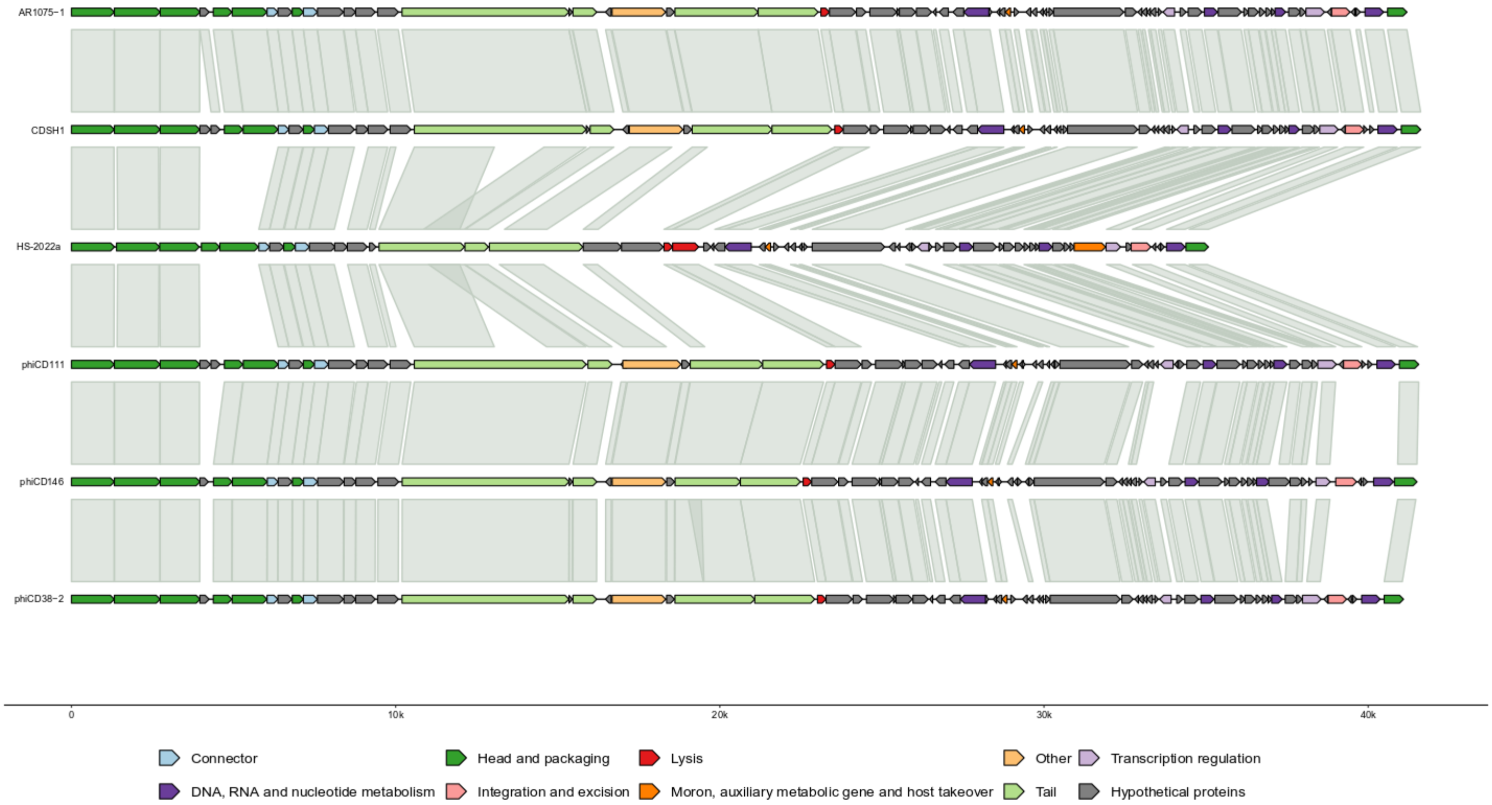

Figure S12: Genome comparison among cluster D phage genomes. This cluster is highly conserved, especially, since the five genomes phiCD38-2, AR1075-1, CDSH1, phiCD111, and phiCD146 show very high levels of similarity which is observed in the map.

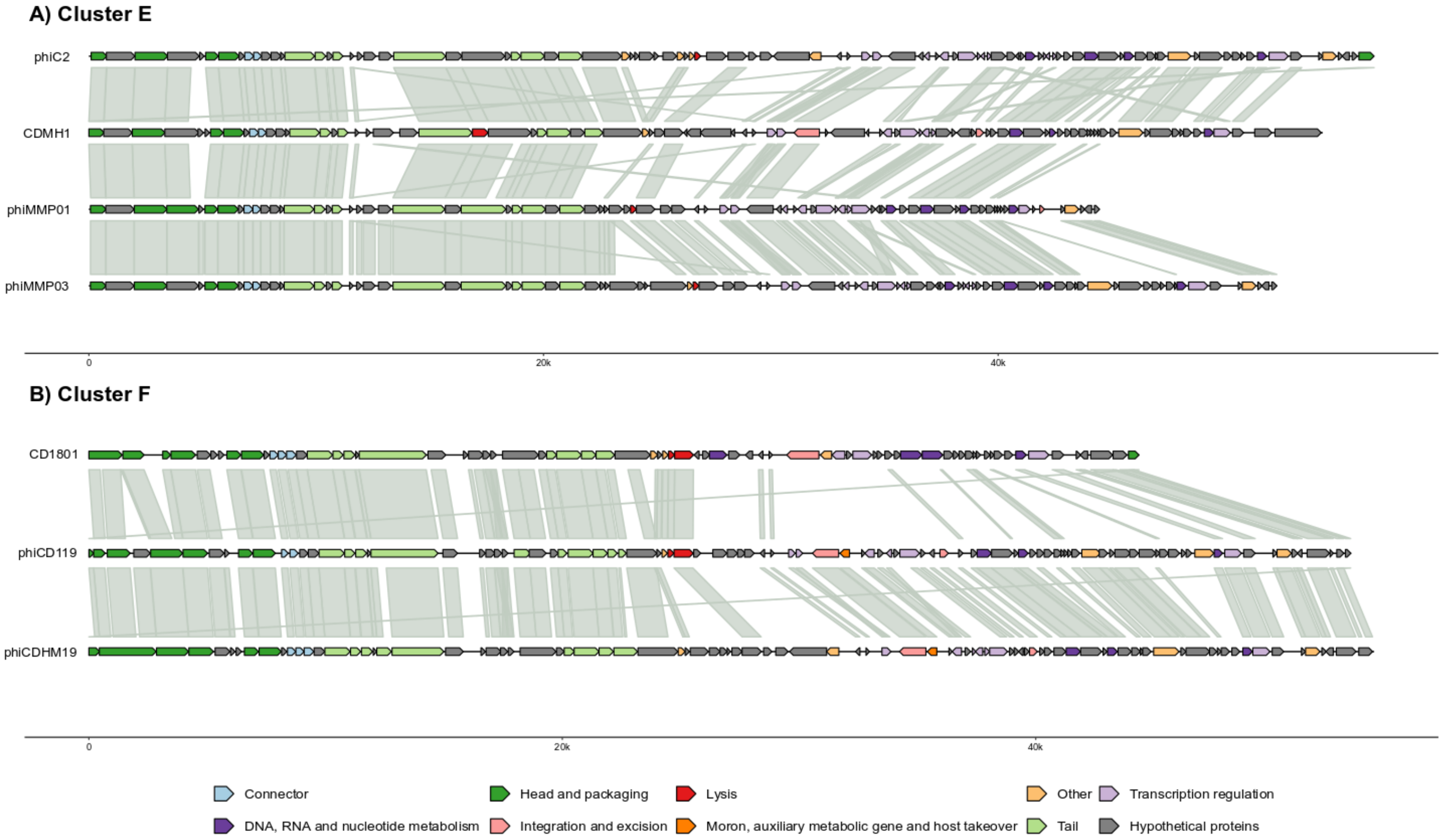

Figure S13: Genome comparison among cluster E **(A)** and F **(B)** phage genomes. Similar to cluster D, three genomes of cluster E are highly conserved compared to the fourth phage genome CDMH1.

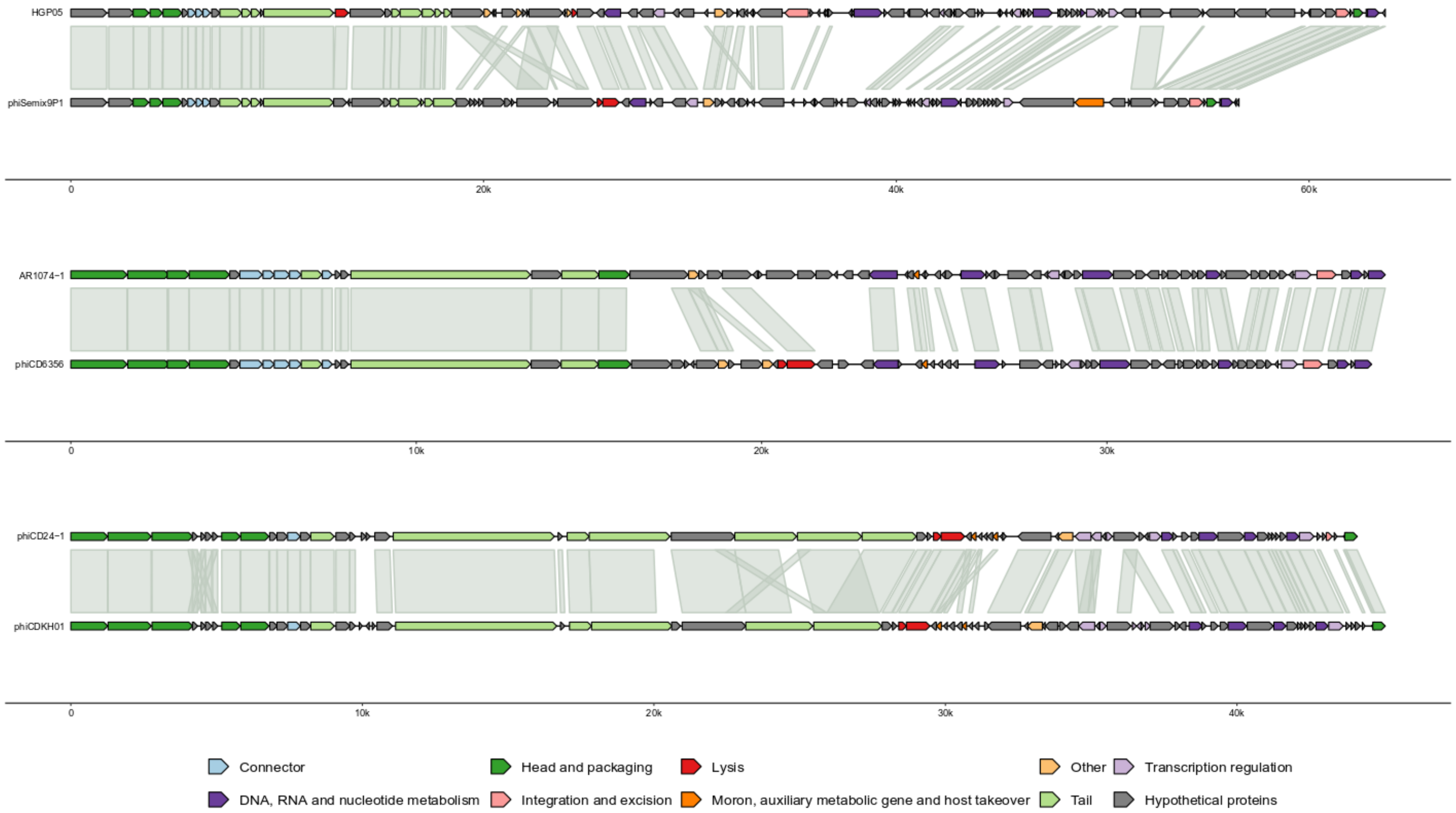

Figure S14: Genome comparison among cluster G **(A)**, H **(B)**, and I **(C)** phage genomes.

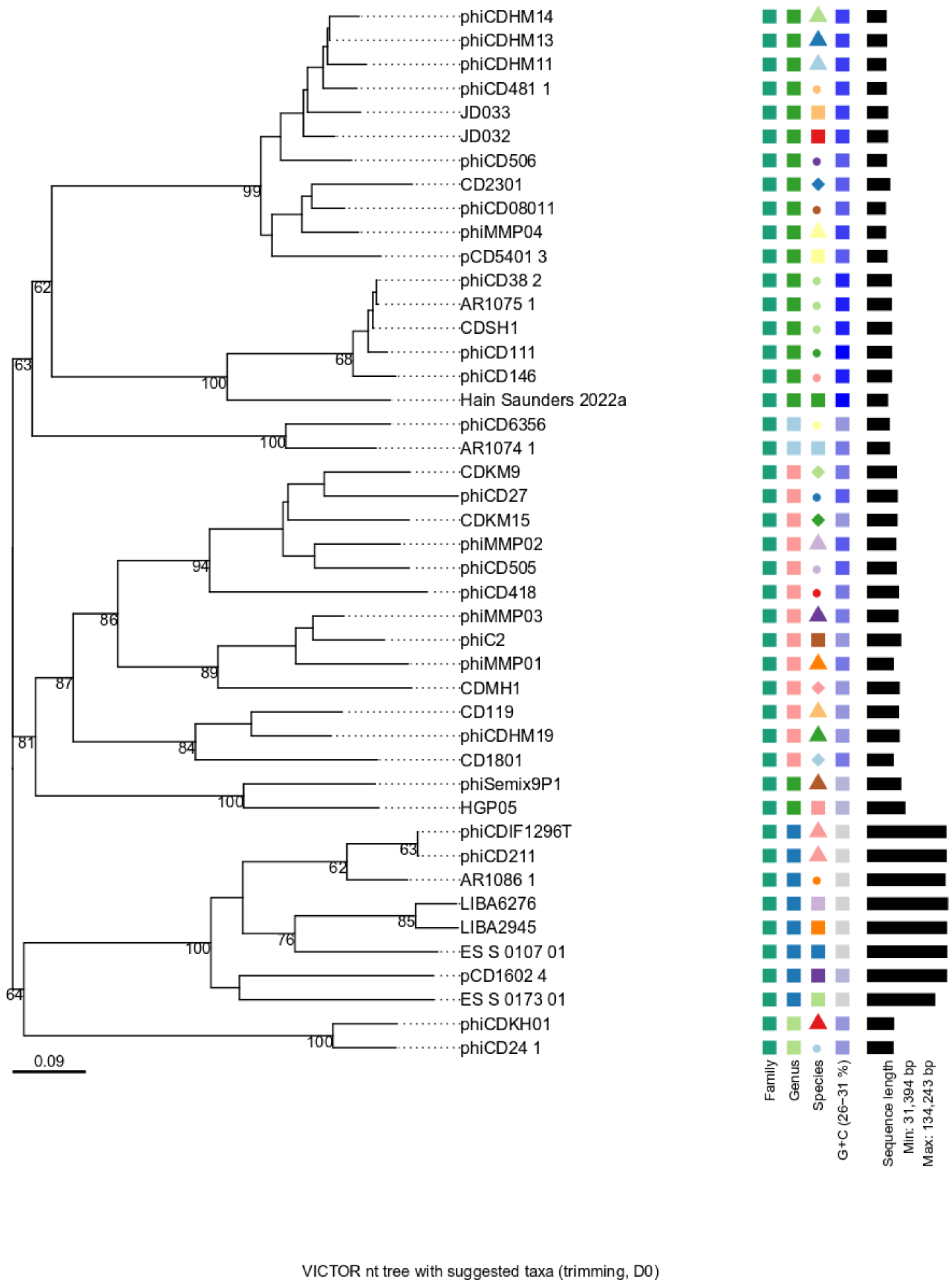

Figure S15: Phylogenomic GBDP tree generated using VICTOR webserver with suggested taxa. The tree depicts 41 species-level clusters, five genus-level clusters, and one family-level cluster. Members of the same clusters are indicated by the same color. GC percentages and genome length of each phage are also represented in the tree.

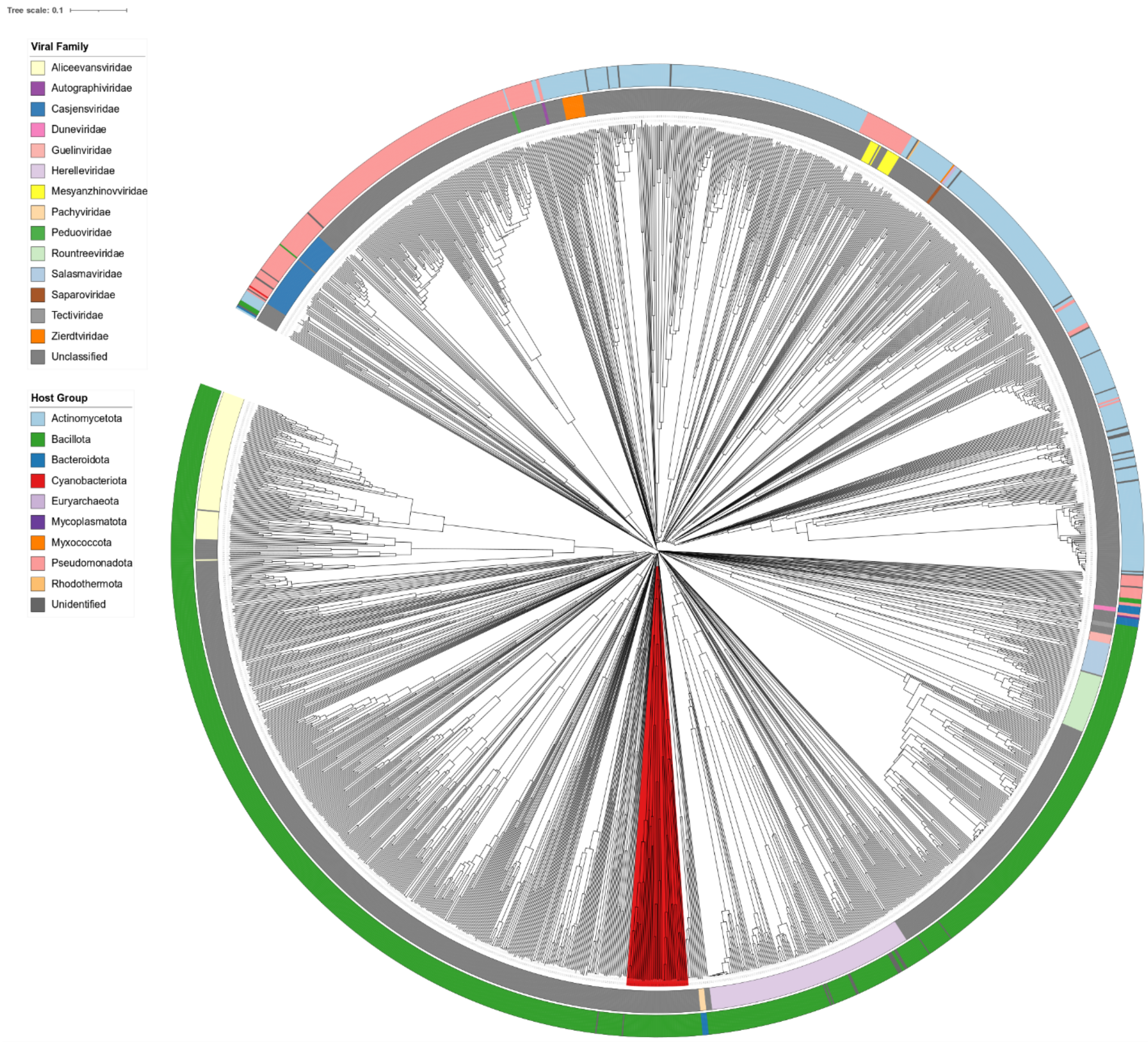

Figure S16: Proteomic tree generated by ViPtree using CD phage and reference sequences. The inner color strip represents the viral family of the phages and the outer strip represents the phylum of their respective bacterial host. CD phages are marked as red in the tree and form a separate clade with no close relative.

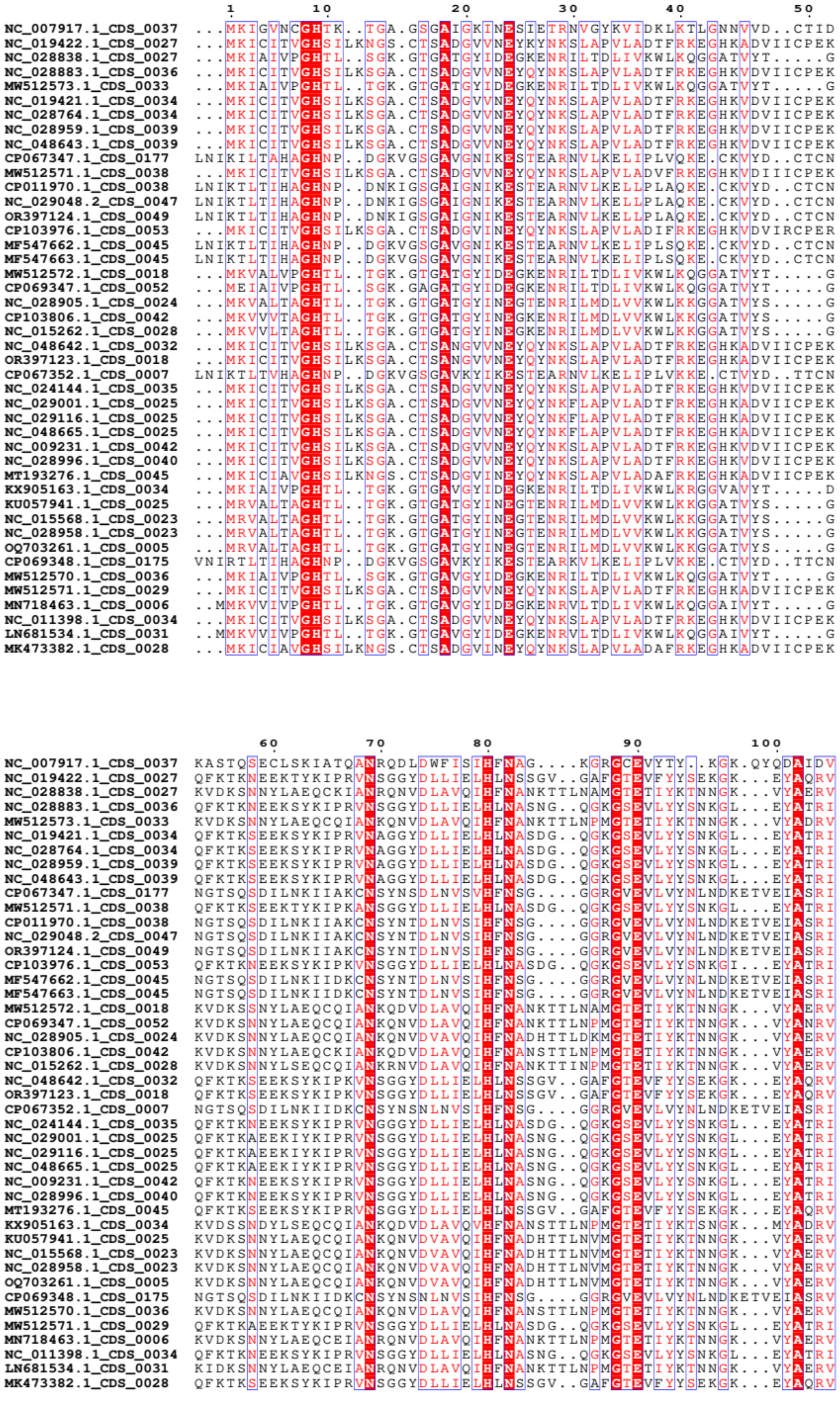
1 10 20 30 40 50
NC_007917.1_CDS_0037 ...MKIGVNCGHTK..TGA.GSGAIGKINESIETRNVGYKVIDKLKTLGNNVVD..CTID
NC_019422.1_CDS_0027 ...MKICITVGHSILKNGS.CTSADGVVNEYKYNKSLAPVLADTFRKEGHKADVIICPEK
NC_028838.1_CDS_0027 ...MKIAIVPGHTL..SGK.GTGATGYIDEGKENRILTDLIVKWLKQGGATVYT.....G
NC_028883.1_CDS_0036 ...MKICITVGHSILKSGA.CTSADGVVNEYQYNKSLAPVLADTFRKEGHKVDVIICPEK
MW512573.1_CDS_0033 ...MKIAIVPGHTL..TGK.GTGATGYIDEGKENRILTDLIVKWLKQGGATVYT.....G
NC_019421.1_CDS_0034 ...MKICITVGHSILKSGA.CTSADGVVNEYQYNKSLAPVLADTFRKEGHKVDVIICPEK
NC_028764.1_CDS_0034 ...MKICITVGHSILKSGA.CTSADGVVNEYQYNKSLAPVLADTFRKEGHKVDVIICPEK
NC_028959.1_CDS_0039 ...MKICITVGHSILKSGA.CTSADGVVNEYQYNKSLAPVLADTFRKEGHKVDVIICPEK
NC_048643.1_CDS_0039 ...MKICITVGHSILKSGA.CTSADGVVNEYQYNKSLAPVLADTFRKEGHKVDVIICPEK
CP067347.1_CDS_0177 LNIKILTAHAGHNP..DGKVGSGAVGNIKESTEARNVLKELIPLVQKE.CKVYD..CTCN
MW512571.1_CDS_0038 ...MKICITVGHSILKSGA.CTSADGVVNEYQYNKSLAPVLADVFRKEGHKVDIIICPEK
CP011970.1_CDS_0038 LNIKTLTIHAGHNP..DNKIGSGAIGNIKESTEARNVLKELLPLAQKE.CKVYD..CTCN
NC_029048.2_CDS_0047 LNIKTLTIHAGHNP..DNKIGSGAIGNIKESTEARNVLKELLPLAQKE.CKVYD..CTCN
OR397124.1_CDS_0049 LNIKTLTIHAGHNP..DNKIGSGAIGNIKESTEARNVLKELLPLAQKE.CKVYD..CTCN
CP103976.1_CDS_0053 ...MKICITVGHSILKSGA.CTSADGVINEYQYNKSLAPVLADIFRKEGHKVDVIRCPER
MF547662.1_CDS_0045 LNIKTLTIHAGHNP..DGKVGSGAVGNIKESTEARNVLKELIPLSQKE.CKVYD..CTCN
MF547663.1_CDS_0045 LNIKTLTIHAGHNP..DGKVGSGAVGNIKESTEARNVLKELIPLSQKE.CKVYD..CTCN
MW512572.1_CDS_0018 ...MKVALVPGHTL..TGK.GTGATGYIDEGKENRILTDLIVKWLKQGGATVYT.....G
CP069347.1_CDS_0052 ...MEIAIVPGHTL..SGK.GAGATGYIDEGKENRILTDLIVKWLKQGGATVYT.....G
NC_028905.1_CDS_0024 ...MKVALTAGHTL..TGK.GTGATGYINEGTENRILMDLVVKWLKKGGATVYS.....G
CP103806.1_CDS_0042 ...MKVVVTAGHTL..TGK.GTGATGYINEGKENRILMDLVVKWLKKGGATVYS.....G
NC_015262.1_CDS_0028 ...MKVVLTAGHTL..TGK.GTGATGYINEGKENRILMDLVVKWLKKGGATVYS.....G
NC_048642.1_CDS_0032 ...MKICITVGHSILKSGA.CTSANGVVNEYQYNKSLAPVLADTFRKEGHKADVIICPEK
OR397123.1_CDS_0018 ...MKICITVGHSILKSGA.CTSANGVVNEYQYNKSLAPVLADTFRKEGHKADVIICPEK
CP067352.1_CDS_0007 LNIKTLTVHAGHNP..DGKVGSGAVKYIKESTEARNVLKELIPLVKKE.CTVYD..TTCN
NC_024144.1_CDS_0035 ...MKICITVGHSILKSGA.CTSADGVVNEYQYNKSLAPVLADTFRKEGHKVDVIICPEK
NC_029001.1_CDS_0025 ...MKICITVGHSILKSGA.CTSADGVVNEYQYNKFLAPVLADTFRKEGHKADVIICPEK
NC_029116.1_CDS_0025 ...MKICITVGHSILKSGA.CTSADGVVNEYQYNKFLAPVLADTFRKEGHKADVIICPEK
NC_048665.1_CDS_0025 ...MKICITVGHSILKSGA.CTSADGVVNEYQYNKFLAPVLADTFRKEGHKADVIICPEK
NC_009231.1_CDS_0042 ...MKICITVGHSILKSGA.CTSADGVVNEYQYNKSLAPVLADTFRKEGHKVDVIICPEK
NC_028996.1_CDS_0040 ...MKICITVGHSILKSGA.CTSADGVVNEYQYNKSLAPVLADTFRKEGHKVDVIICPEK
MT193276.1_CDS_0045 ...MKICITVGHSILKNGS.CTSADGVINEYQYNKSLAPVLADAFRKEGHKADVIICPEK
KX905163.1_CDS_0034 ...MKIAIVPGHTL..TGK.GTGAVGYIDEGKENRILTDLIVKWLKRGGVAVYT.....D
KU057941.1_CDS_0025 ...MRVALTAGHTL..TGK.GTGATGYINEGTENRILMDLVVKWLKKGGATVYS.....G
NC_015568.1_CDS_0023 ...MRVALTAGHTL..TGK.GTGATGYINEGTENRILMDLVVKWLKKGGATVYS.....G
NC_028958.1_CDS_0023 ...MRVALTAGHTL..TGK.GTGATGYINEGTENRILMDLVVKWLKKGGATVYS.....G
OQ703261.1_CDS_0005 ...MRVALTAGHTL..TGK.GTGATGYINEGTENRILMDLVVKWLKKGGATVYS.....G
CP069348.1_CDS_0175 VNIRTLTIHAGHNP..DGKVGSGAVKYIKESTEARKVLKELIPLVKKE.CTVYD..TTCN
MW512570.1_CDS_0036 ...MKIAIVPGHTL..SGK.GTGAVGYIDEGKENRILTDLIVKWLKQGGATVYT.....G
MW512571.1_CDS_0029 ...MKICITVGHSILKSGA.CTSADGVVNEYQYNKSLAPVLADTFRKEGHKADVIICPEK
MN718463.1_CDS_0006 ..MMKVVIVPGHTL..TGK.GTGAVGYIDEGKENRVLTDLIVKWLKQGGAIVYT.....G
NC_011398.1_CDS_0034 ...MKICITVGHSILKSGA.CTSADGVVNEYQYNKSLAPVLADTFRKEGHKVDVIICPEK
LN681534.1_CDS_0031 ..MMKVVIVPGHTL..TGK.GTGAVGYIDEGKENRVLTDLIVKWLKQGGAIVYT.....G
MK473382.1_CDS_0028 ...MKICIAVGHSILKNGS.CTSADGVINEYQYNKSLAPVLADAFRKEGHKADVIICPEK
60 70 80 90 100
NC_007917.1_CDS_0037 KASTQSECLSKIATQANRQDLDWFISIHFNAG....KGRGCEVYTY..KGK.QYQDAIDV
NC_019422.1_CDS_0027 QFKTKNEEKTYKIPRVNSGGYDLLIELHLNSSGV..GAFGTEVFYYSEKGK...EYAQRV
NC_028838.1_CDS_0027 KVDKSNNYLAEQCKIANRQNVDLAVQIHFNANKTTLNAMGTETIYKTNNGK...VYAERV
NC_028883.1_CDS_0036 QFKTKNEEKSYKIPRVNSGGYDLLIELHLNASNG..QGKGSEVLYYSNKGL...EYATRI
MW512573.1_CDS_0033 KVDKSNNYLAEQCQIANKQNVDLAVQIHFNANKTTLNPMGTETIYKTNNGK...VYADRV
NC_019421.1_CDS_0034 QFKTKSEEKSYKIPRVNAGGYDLLIELHLNASDG..QGKGSEVLYYSNKGL...EYATRI
NC_028764.1_CDS_0034 QFKTKSEEKSYKIPRVNAGGYDLLIELHLNASDG..QGKGSEVLYYSNKGL...EYATRI
NC_028959.1_CDS_0039 QFKTKSEEKSYKIPRVNAGGYDLLIELHLNASDG..QGKGSEVLYYSNKGL...EYATRI
NC_048643.1_CDS_0039 QFKTKSEEKSYKIPRVNAGGYDLLIELHLNASDG..QGKGSEVLYYSNKGL...EYATRI
CP067347.1_CDS_0177 NGTSQSDILNKIIAKCNSYNSDLNVSVHFNSG....GGRGVEVLVYNLNDKETVEIASRI
MW512571.1_CDS_0038 QFKTKSEEKTYKIPKANSGGYDLLIELHLNASDG..QGKGSEVLYYSNKGL...EYATRI
CP011970.1_CDS_0038 NGTSQSDILNKIIAKCNSYNTDLNVSIHFNSG....GGRGVEVLVYNLNDKETVEIASRI
NC_029048.2_CDS_0047 NGTSQSDILNKIIAKCNSYNTDLNVSIHFNSG....GGRGVEVLVYNLNDKETVEIASRI
OR397124.1_CDS_0049 NGTSQSDILNKIIAKCNSYNTDLNVSIHFNSG....GGRGVEVLVYNLNDKETVEIASRI
CP103976.1_CDS_0053 QFKTKNEEKSYKIPKVNSGGYDLLIELHLNASDG..QGKGSEVLYYSNKGI...EYATRI
MF547662.1_CDS_0045 NGTSQSDILNKIIDKCNSYNTDLNVSIHFNSG....GGRGVEVLVYNLNDKETVEIASRI
MF547663.1_CDS_0045 NGTSQSDILNKIIDKCNSYNTDLNVSIHFNSG....GGRGVEVLVYNLNDKETVEIASRI
MW512572.1_CDS_0018 KVDKSSNYLAEQCQIANKQDVDLAVQIHFNANKTTLNAMGTETIYKTNNGK...VYAERV
CP069347.1_CDS_0052 KVDKSNNYLAEQCQIANKQNVDLAVQIHFNANKTTLNPMGTETIYKTNNGK...VYANRV
NC_028905.1_CDS_0024 KVDKSNNYLAEQCQIANKQNVDVAVQIHFNADHTTLDKMGTETIYKTNNGK...VYAERV
CP103806.1_CDS_0042 KVDKSNNYLAEQCKIANKQDVDLAVQIHFNANSTTLNPMGTETIYKTNNGK...VYAERV
NC_015262.1_CDS_0028 KVDKSNNYLSEQCQIANKRNVDLAVQIHFNANKTTINPMGTETIYKTNNGK...VYAERV
NC_048642.1_CDS_0032 QFKTKSEEKSYKIPKVNSGGYDLLIELHLNSSGV..GAFGTEVFYYSEKGK...EYAQRV
OR397123.1_CDS_0018 QFKTKSEEKSYKIPKVNSGGYDLLIELHLNSSGV..GAFGTEVFYYSEKGK...EYAQRV
CP067352.1_CDS_0007 NGTSQSDILNKIIDKCNSYNSNLNVSIHFNSG....GGRGVEVLVYNLNDKETVEIASRI
NC_024144.1_CDS_0035 QFKTKNEEKSYKIPRVNGGGYDLLIELHLNASDG..QGKGSEVLYYSNKGL...EYATRI
NC_029001.1_CDS_0025 QFKTKAEEKIYKIPRVNSGGYDLLIELHLNASNG..QGKGSEVLYYSNKGL...EYATRI
NC_029116.1_CDS_0025 QFKTKAEEKIYKIPRVNSGGYDLLIELHLNASNG..QGKGSEVLYYSNKGL...EYATRI
NC_048665.1_CDS_0025 QFKTKAEEKIYKIPRVNSGGYDLLIELHLNASNG..QGKGSEVLYYSNKGL...EYATRI
NC_009231.1_CDS_0042 QFKTKNEEKSYKIPRVNSGGYDLLIELHLNASNG..QGKGSEVLYYSNKGL...EYATRI
NC_028996.1_CDS_0040 QFKTKNEEKSYKIPRVNSGGYDLLIELHLNASNG..QGKGSEVLYYSNKGL...EYATRI
MT193276.1_CDS_0045 QFKTKSEEKSYKIPRVNSGGYDLLIELHLNSSGV..GAFGTEVFYYSEKGK...EYAQRV
KX905163.1_CDS_0034 KVDSSNDYLSEQCQIANKQDVDLAVQVHFNANSTTLNPMGTETIYKTSNGK...MYADRV
KU057941.1_CDS_0025 KVDKSNNYLAEQCQIANKQNVDVAVQIHFNADHTTLNVMGTETIYKTNNGK...VYAERV
NC_015568.1_CDS_0023 KVDKSNNYLAEQCQIANKQNVDVAVQIHFNADHTTLNVMGTETIYKTNNGK...VYAERV
NC_028958.1_CDS_0023 KVDKSNNYLAEQCQIANKQNVDVAVQIHFNADHTTLNVMGTETIYKTNNGK...VYAERV
OQ703261.1_CDS_0005 KVDKSNNYLAEQCQIANKQNVDVAVQIHFNADHTTLNVMGTETIYKTNNGK...VYAERV
CP069348.1_CDS_0175 NGTSQSDILNKIIDKCNSYNSNLNVSIHFNSG....GGRGVEVLVYNLNDKETVEIASRV
MW512570.1_CDS_0036 KVDKSNNYLAEQCQIANKQNVDLAVQIHFNANSTTLNPMGTETIYKTNNGK...VYAERV
MW512571.1_CDS_0029 QFKTKAEEKTYKIPRVNSGGYDLLIELHLNASDG..QGKGSEVLYYSNKGL...EYATRI
MN718463.1_CDS_0006 KVDKSNNYLAEQCEIANRQNVDLAVQIHFNANKTTLNPMGTETIYKTNNGK...VYAERV
NC_011398.1_CDS_0034 QFKTKNEEKSYKIPRVNSGGYDLLIELHLNASNG..QGKGSEVLYYSNKGL...EYATRI
LN681534.1_CDS_0031 KIDKSNNYLAEQCEIANRQNVDLAVQIHFNANKTTLNPMGTETIYKTNNGK...VYAERV
MK473382.1_CDS_0028 QFKTKSEEKSYKIPRVNSGGYDLLIELHLNSSGV..GAFGTEVFYYSEKGK...EYAQRV

```
                                  110       120       130       140       150
NC_007917.1_CDS_0037    CKKISDLG.......FTNRGVKDG.SGLYVVKKTKAKSMLIEVCFVDSEDANKYLSL.GA
NC_019422.1_CDS_0027    VDKLSKPFRKKGDKEVGNRGVKLD.KGLYILNSSKPTAILIESFFCDNKEDYEKAKKFGY
NC_028838.1_CDS_0027    NDKLATVF........ENRGAKSDVRGLYWLSHTKAPAILIEVCFVDSKADTDYYIR.HK
NC_028883.1_CDS_0036    CDKLGTVF........KNRGAKLD.KRLYILNSSKPTAVLIESFFCDNKEDYDKAKKLGH
MW512573.1_CDS_0033     NTKLATVF........KNRGAKSDVRGLYWLSHTKAPAILIEVCFVDSKADTDYYIK.NK
NC_019421.1_CDS_0034    CKKLGTIF........KNRGAKLD.KGLYILNSSKPTAILIESFFCDNKDDYEKAKKFGY
NC_028764.1_CDS_0034    CKKLGTIF........KNRGAKLD.KGLYILNSSKPTAILIESFFCDNKDDYEKAKKFGY
NC_028959.1_CDS_0039    CKKLGTIF........KNRGAKLD.KGLYILNSSKPTAILIESFFCDNKDDYEKAKKFGY
NC_048643.1_CDS_0039    CKKLGTIF........KNRGAKLD.KGLYILNSSKPTAILIESFFCDNKDDYEKAKKFGY
CP067347.1_CDS_0177     CEQITKTYRTKGDKAFKNRGVKEK.KTLAFLRRTKAKSLLVECCFVDTDDTKNY....NA
MW512571.1_CDS_0038     CKKLGTVF........KNRGAKLD.KGLYILNSSKPTAVLIESFFCDNKEDYEKAKKLGH
CP011970.1_CDS_0038     CKKITETYHAKGDKDFKNRGVKEK.KTLAFLRRTKAKSILVECCFVDTSDTKKY....NA
NC_029048.2_CDS_0047    CKKITETYHAKGDKDFKNRGVKEK.KTLAFLRRTKAKSILVECCFVDTSDTKKY....NA
OR397124.1_CDS_0049     CKKITETYHAKGDKDFKNRGVKEK.KTLAFLRRTKAKSILVECCFVDTSDTKKY....NA
CP103976.1_CDS_0053     CKKLGTVF........KNRGAKLD.KGLYILNSSKPTAVLIESFFCDNKEDYEKAKKLGY
MF547662.1_CDS_0045     CKKITETYHAKGDKDFKNRGVKEK.KTLAFLRRTKAKSILVECCFVDTSDTKKY....NA
MF547663.1_CDS_0045     CKKITETYHAKGDKDFKNRGVKEK.KTLAFLRRTKAKSILVECCFVDTSDTKKY....NA
MW512572.1_CDS_0018     NTKLATVF........KNRGAKSDARGLYWLSHTKAPAILIEVCFVDSKADTDYYIR.HK
CP069347.1_CDS_0052     NDKLATVF........KNRGAKSDTRGLYWLNHTKAPAILIEVCFVDSKADTDYYIR.HK
NC_028905.1_CDS_0024    NEKLATIF........KNRGAKSDARGLYWLSHTKAPAILIEVCFVDSKADTDYYIR.HK
CP103806.1_CDS_0042     NKKLSTIF........KNRGAKSDVRGLYWLSHTKAPAILIEVCFVDSKADTDYYIR.HK
NC_015262.1_CDS_0028    NEKLATVF........ENRGAKSDARGLYWLRHTKAPAILIEVCFVDSKADTDYYIR.HK
NC_048642.1_CDS_0032    VDKLSKPFRKKGDKEVGNRGVKLD.KSLYILNSSKPTAILIESFFCDNKEDYEKAKKLGH
OR397123.1_CDS_0018     VDKLSKPFRKKGDKEVGNRGVKLD.KSLYILNSSKPTAILIESFFCDNKEDYEKAKKLGH
CP067352.1_CDS_0007     CEKITKTYHAKGDKSFKNRGVKEK.KTLAFLRRTKAKSILVECCFVDTDDTKNY....NA
NC_024144.1_CDS_0035    CNKLGTVF........RNRRAKLD.KGLYILNSSNPTAVLIESFFCDNKEDYEKAKKLDH
NC_029001.1_CDS_0025    CNKLGTVF........RNRRAKLD.KGLYILNSSKPTAVLIESFFCDNKEDYEKAKKLGY
NC_029116.1_CDS_0025    CNKLGTVF........RNRRAKLD.KGLYILNSSKPTAVLIESFFCDNKEDYEKAKKLGY
NC_048665.1_CDS_0025    CNKLGTVF........RNRRAKLD.KGLYILNSSKPTAVLIESFFCDNKEDYEKAKKLGY
NC_009231.1_CDS_0042    CDKLGTVF........KNRGAKLD.KRLYILNSSKPTAVLIESFFCDNKEDYDKAKKLGH
NC_028996.1_CDS_0040    CDKLGTVF........KNRGAKLD.KRLYILNSSKPTAVLIESFFCDNKEDYDKAKKLGH
MT193276.1_CDS_0045     VDKLSKPFRKKGDKEVGNRGVKLD.KSLYILNSSKPTAVLIESFFCDNKEDYEKAKKLGY
KX905163.1_CDS_0034     NDKLATVF........KNRGAKSDVIGLYWLSHTKAPAILIEVCFVDSKADTDYYIR.HK
KU057941.1_CDS_0025     NEKLATIF........KNRGAKSDARGLYWLSHTKAPAILIEVCFVDSKADTDYYIR.HK
NC_015568.1_CDS_0023    NEKLATIF........KNRGAKSDARGLYWLSHTKAPAILIEVCFVDSKADTDYYIR.HK
NC_028958.1_CDS_0023    NEKLATIF........KNRGAKSDARGLYWLSHTKAPAILIEVCFVDSKADTDYYIR.HK
OQ703261.1_CDS_0005     NEKLATIF........KNRGAKSDARGLYWLSHTKAPAILIEVCFVDSKADTDYYIR.HK
CP069348.1_CDS_0175     CKKITETYHAKGDKDFKNRGVKEK.KTLAFLRRTKAKSILVECCFVDTDDTKNY....NA
MW512570.1_CDS_0036     NDKLATVF........KNRGAKSDVRGLYWLSHTKAPAILIEVCFVDSKADTDYYIR.HK
MW512571.1_CDS_0029     CNKLGTVF........RNRRAKLD.KGLYILNSSNPTAVLIESFFCDNKEDYEKAKKLGH
MN718463.1_CDS_0006     NKKLSTIF........KNRGAKSDVRGLYWLSHTKAPAILIEVCFVDSKADTDYYIR.HK
NC_011398.1_CDS_0034    CDKLGTVF........KNRGAKLD.KRLYILNSSKPTAVLIESFFCDNKEDYDKAKKLGH
LN681534.1_CDS_0031     NKKLSTIF........KNRGAKSDVRGLYWLSHTKAPAILIEVCFVDSKADTDYYIR.HK
MK473382.1_CDS_0028     VDKLSKPFRKKGDKEVGNRGVKLD.KSLYILNSSKPTAVLIESFFCDNKEDYEKAKKLGY
```

```
                         160       170       180       190       200       210
NC_007917.1_CDS_0037    DKLATAIVEAI.TKHISSAE..ENNYNRYKHTIVYSGD.DKVSADILGLYYKRKKYLVTD
NC_019422.1_CDS_0027    EGIAKLIVEGILNKNINNKEDSEG.KIMYKHTIVYDGEVDKIPATVVGWGYNDGKILICD
NC_028838.1_CDS_0027    DIVAKLIAEGILNKTIDNKENGEG.KIMYKHTIVYDGEVDKIPATVVGWGYNDGKILICD
NC_028883.1_CDS_0036    EGIAKLIVEGVLNKNINN....EGVKQMYKHTIVYDGEVDKIPATVVGWGYNDGKILICD
MW512573.1_CDS_0033     NTVAKLIAEGILNKKIDNIEV....KQMYKHTVIYEGEVDKVLAQIISWNYKENECRVCD
NC_019421.1_CDS_0034    EGMAKLIVEGVLNKNIGN....DGVKLMYKHTIVYDGEVDKIPATVVGWGYNDGKILICD
NC_028764.1_CDS_0034    EGMAKLIVEGVLNKNIGN....DGVKLMYKHTIVYDGEVDKIPATVVGWGYNDGKILICD
NC_028959.1_CDS_0039    EGMAKLIVEGVLNKNIGN....DGVKLMYKHTIVYDGEVDKIPATVVGWGYNDGKILICD
NC_048643.1_CDS_0039    EGMAKLIVEGVLNKNIGN....DGVKLMYKHTIVYDGEVDKIPATVVGWGYNDGKILICD
CP067347.1_CDS_0177     KDMAIDIAEGIFNKSIK.....QTQEEKMKYTIVYEGEVDKAIANVMAINYKSDEVYVCE
MW512571.1_CDS_0038     EGIAKLIVEGVLNKNINS....EGVKQMYKHTIVYDGEVDKIPATVVGWGYNDGKILICD
CP011970.1_CDS_0038     KDMAIDIYEGIFNKSVAG....KPQDNKVKYAIVYEGDVDKVIAQLMAMNYKTNEVSVCD
NC_029048.2_CDS_0047    KDMAIDIYEGIFNKSVAG....KPQDNKVKYAIVYEGDVDKVIAQLMAMNYKTNEVSVCD
OR397124.1_CDS_0049     KDMAIDIYEGIFNKSVAG....KPQDNKVKYAIVYEGDVDKVIAQLMAMNYKTNEVSVCD
CP103976.1_CDS_0053     EGIAKLIVEGVLNKNISS....EGVKQMYKHTIIYDGEIDKIPATVVGWGYKNGKILICD
MF547662.1_CDS_0045     KDMAIDIYEGIFNKSVAG....KPQDNKVKYAIVYEGEVDKVIAQLMAMNYKSNEVSICE
MF547663.1_CDS_0045     KDMAIDIYEGIFNKSVAG....KPQDNKVKYAIVYEGEVDKVIAQLMAMNYKSNEVSICE
MW512572.1_CDS_0018     DIVAKLIAEGILNKNINN....EGVKQMYKHTIVYDGEVDKIPATVVGWGYNDGKILICD
CP069347.1_CDS_0052     DIIAKLIAEGILNKKIDN....EV.KQMYKHTIVYDGEVDKIPATVVGWGYSDGKIFICD
NC_028905.1_CDS_0024    DIVAKLIAEGILNKTIDNKENSED.KKMYKHTIVYDGEVDKIPATVVGWGYNDGKILICD
CP103806.1_CDS_0042     DIAAKLIAEGILNKTIDNKENGEG.KIMYKHTIVYDGAVDKITATVVGWGYNDGKILICD
NC_015262.1_CDS_0028    DIVAKLIAEGILNKNIDNKENGED.KKMYKHTIVYDGEVDKILATVLGWGYSSSKVLVCD
NC_048642.1_CDS_0032    EGIAKLIVEGVLNKNINN....EGVKQMYKHTIIYDGEVDKIPATVVGWGYNDGKILICD
OR397123.1_CDS_0018     EGIAKLIVEGVLNKNINN....EGVKQMYKHTIIYDGEVDKIPATVVGWGYNDGKILICD
CP067352.1_CDS_0007     KDMATDIAEGIFNKSIN.....QTQEEKMKYTIVYEGEVDKAIANVMAINYKSDEVYVCE
NC_024144.1_CDS_0035    EGIAKLIVEGVLNKNINN....EGVKQMYKHTIVYDGEVDKIPATVVGWGYNDGKILICD
NC_029001.1_CDS_0025    EGMAKLIVEGVLNKNIGD....EGVKEMYKHTIVYSGEVDKIPATVVGWGYNDGKILICD
NC_029116.1_CDS_0025    EGMAKLIVEGVLNKNIGD....EGVKEMYKHTIVYSGEVDKIPATVVGWGYNDGKILICD
NC_048665.1_CDS_0025    EGMAKLIVEGVLNKNIGD....EGVKEMYKHTIVYSGEVDKIPATVVGWGYNDGKILICD
NC_009231.1_CDS_0042    EGIAKLIVEGVLNKNINN....EGVKQMYKHTIVYDGEVDKILANVLSWGYSPSKVLVCD
NC_028996.1_CDS_0040    EGIAKLIVEGVLNKNINN....EGVKQMYKHTIVYDGEVDKILANVLSWGYSPSKVLVCD
MT193276.1_CDS_0045     EGIAKLIVEGVLNKNIGD....EGVKEMYKHTIVYSGEVDKIPATVVGWGYNDGKILICD
KX905163.1_CDS_0034     DIVAKLIAEGILNKKIDSEEV....NKMYKHTIVYDGEIDKIPATVVGWGYNNGKILICD
KU057941.1_CDS_0025     DIVAKLIAEGILNKTIDNKENSED.KKMYKHTIVYDGEVDKIPATVVGWGYNDGKILICD
NC_015568.1_CDS_0023    DIVAKLIAEGILNKTIDNKENSED.KKMYKHTIVYDGEVDKIPATVVGWGYNDGKILICD
NC_028958.1_CDS_0023    DIVAKLIAEGILNKTIDNKENSED.KKMYKHTIVYDGEVDKIPATVVGWGYNDGKILICD
OQ703261.1_CDS_0005     DIVAKLIAEGILNKTIDNKENSED.KKMYKHTIVYDGEVDKIPATVVGWGYNDGKILICD
CP069348.1_CDS_0175     KDMAINIFEGIFNKSVSG....SSQDKKNKYTIVYEGEVDKAIANVMAINYKSDEVYVCE
MW512570.1_CDS_0036     DIVAKLIAEGILNKNINN....EGVKQMYKHTIVYDGEVDKIPATVVGWGYNDGKILICD
MW512571.1_CDS_0029     EGIAKLIVEGVLNKNINN....EGVKQMYKHTIVYDGEVDKISATVVGWGYNDGKILICD
MN718463.1_CDS_0006     DIVAKLIAEGILNKIIDNKENGEG.KIMYKHTIIYDGAVDKIPATVVGWGYNDGKILICD
NC_011398.1_CDS_0034    EGIAKLIVEGVLNKNINN....EGVKQMYKHTIVYDGEVDKISATVVGWGYNDGKILICD
LN681534.1_CDS_0031     DIVAKLIAEGILNKTIDNKENGEG.KIMYKHTIVYDGAVDKIPATVVGWGYNDGKILICD
MK473382.1_CDS_0028     EGIAKLIVEGVLNKNINS....EGVKQMYKHTIVYDGEIDKIPATVLGWGYGSNKILICD
```

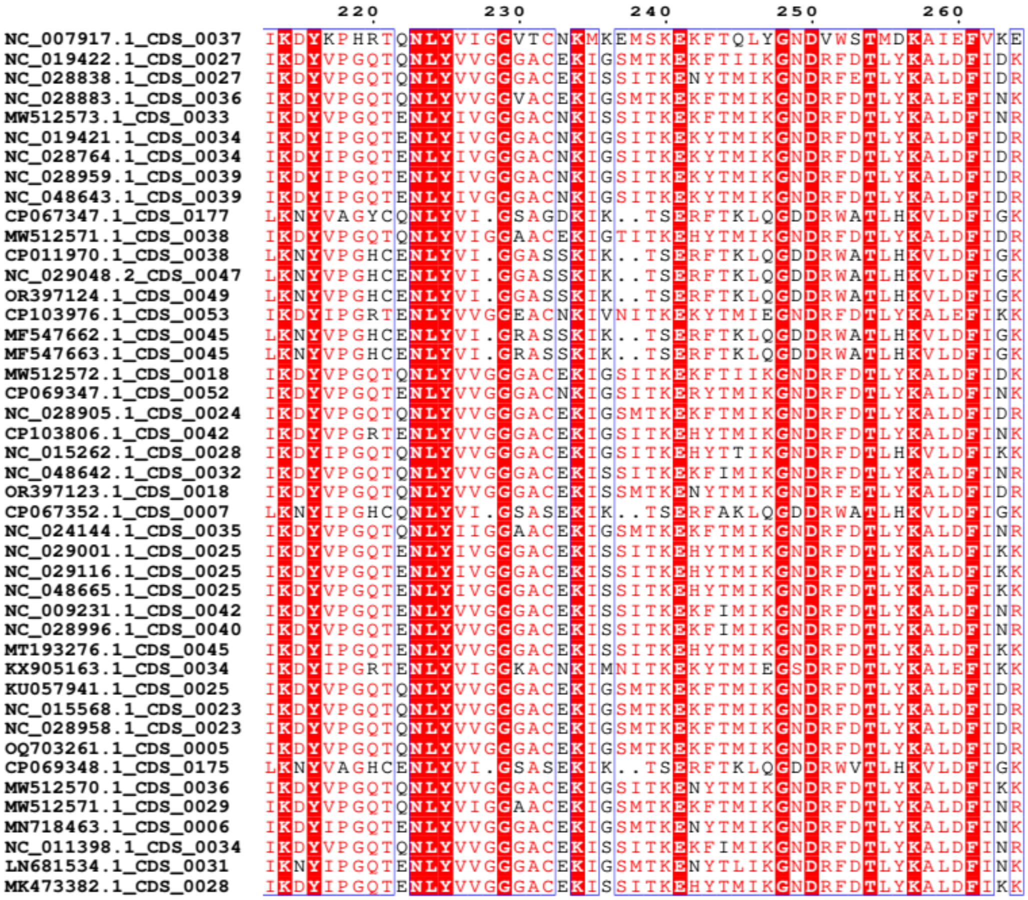

Figure S17: Alignment of amidase_3 containing endolysins. Alignment of amidase_3 endolysins sequences was carried out with MAFFT followed by alignment trimming using trimAl. The resulting alignment was visualized with ESPript 3.0 (https://espript.ibcp.fr/ESPript/ESPript/).

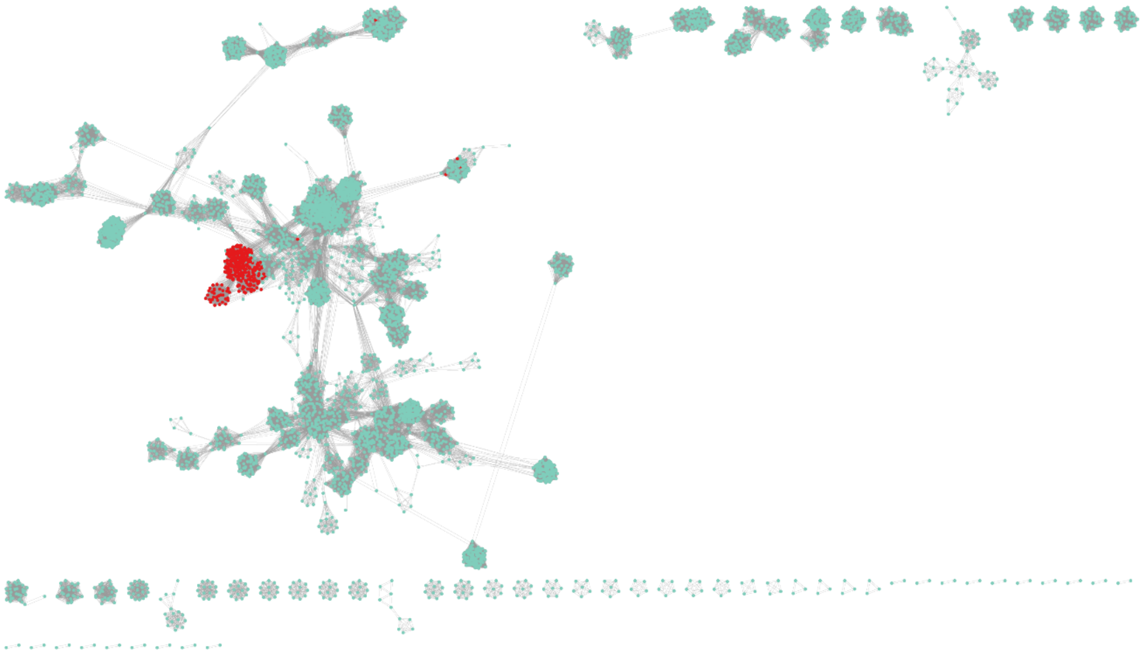

Figure S18: Network file generated in vContact2 and visualized in Cytoscape v3.10.2. *C. difficile* phage genomes both refseq and uncultivated genomes are colored in red. These genomes show high interaction with each other, as evidenced by highly interconnected nodes. Several outliers are observed, notably those similar to other phages, including the gram-positive *Streptococcus* phage Javan630 (MK448997) and several gram-negative phages, such as *Escherichia* phage Ro45lw (MK301532).
